# Adaptative Introgression and Local Adaptation Sweep Through High- and Low-Elevation *Senecio* Species on Mount Etna, Sicily

**DOI:** 10.3390/genes17070778

**Published:** 2026-07-01

**Authors:** Edgar L. Y. Wong, Simon J. Hiscock, Dmitry A. Filatov

**Affiliations:** 1 Department of Biology, University of Oxford, Oxford OX1 3EL, UK; 2 Senckenberg—Leibniz Institution for Biodiversity and Earth System Research, Senckenberg Biodiversity and Climate Research Centre, 60325 Frankfurt am Main, Germany; 3 Oxford Botanic Garden and Arboretum, Oxford OX1 4AZ, UK

**Keywords:** adaptive introgression, local adaptation, selective sweeps, *Senecio*, altitude

## Abstract

Background/Objectives: Closely related species provide an opportunity to study evolutionary processes that underpin new species formation. Speciation driven by adaptation to distinct environments, such as adaptation to high and low elevation in *Senecio aethnensis* and *Senecio chrysanthemifolius*, respectively, on Mount Etna, Sicily, is particularly informative about the interplay between natural selection and ongoing interspecific gene flow. Methods: In this study, we analysed the relative contribution of adaptive introgression and local adaptation in these *Senecio* species. We used genome resequencing with short read sequence data, and analysed patterns of polymorphism within and between the two *Senecio* species. Results: Genome-wide scans for selection identified numerous putative selective sweep regions, many of which overlap with selected regions detected in cline-based analysis in the same system. Our results indicate the prevalence of local over introgressive sweeps, consistent with local adaptation to contrasting environments driving divergence of these species. However, many putative introgressive sweeps were also detected, possibly driven by shared selective forces, such as adaptation to volcanic soils and disease resistance, as indicated by GO terms linked to defence responses for some of the introgressive sweeps. Conclusions: The results suggest that even though the two species are now adapted to different environments, introgression has facilitated their adaptation by sharing adaptive alleles between species, which is likely an important factor for adaptation in closely related species.

## 1. Introduction

Diversifying selection driving population divergence and speciation is the classic Darwinian mechanism of speciation “by means of natural selection” [1]. Identifying targets of selection and inferring the parameters of selection at selected sites is important for understanding adaptation and speciation [2,3,4,5,6]. Ecotonal hybrid zones, such as elevational clines maintained by adaptation to low and high elevations, represent natural laboratories to study how selection leads to speciation (e.g., [7,8,9,10,11]). Selection in hybrid zones is typically studied using cline analysis (e.g., [12,13,14,15,16]). The theory linking cline shapes and selection parameters assumes a certain selection–gene flow equilibrium [13]. Although hybrid zone analyses involving selection and introgression (the transfer of genetic material from one species into another through hybridisation) have been conducted in many systems, open questions remain. For instance, how much adaptation is driven by local species-specific selection and how much of it is “global”, with hybridising species undergoing the same adaptation [17]? Does introgression of alleles across the hybrid zone play a significant role in adaptation? These questions can be addressed in analyses of genome-wide patterns of polymorphism in the species forming a hybrid zone.

Spread and fixation of a new adaptive mutation leads to a so-called “hard” selective sweep (reviewed in [18]). Such sweeps are expected to leave various footprints in DNA polymorphism: (a) a reduction in the level of polymorphism locally [19]; (b) a shift in the site frequency spectrum (SFS) towards high- and low-frequency-derived variants [20], as neutral variants in linkage disequilibrium (LD) with beneficial variants change in frequencies [21]; and (c) a pattern of linkage disequilibrium (LD) that is localised—high LD on each side of the beneficial variant and low LD between the loci on opposite sides of the beneficial variant [21,22]. Adaptation can also proceed from standing variation, with spread of an allele that already existed in the population, resulting in so-called “soft” sweeps [23,24]. The relative importance of soft and hard sweeps is debated in the literature [25]. In this paper, we do not aim to distinguish between soft and hard sweeps, instead assuming that most sweeps we detect are hard or nearly hard (hereafter referred to as “sweeps”) because most approaches have low power to detect soft sweeps due to much subtler footprints of soft compared to hard sweeps in patterns of genetic variation [24].

To examine the pattern of polymorphisms, various neutrality tests can be used (reviewed in [26]). For example, Tajima’s D [27] is expected to be negative in proximity to a sweep (e.g., [28]). To detect recent sweeps, several approaches have been developed, e.g., based on the composite-likelihood-ratio (CLR) test fitting expected to observed SFS [29] or using a combination of summary statistics [30]. However, demographic processes such as population size changes could, in some cases, lead to signatures in DNA polymorphism similar to that of selective sweeps, and care should be taken in interpretation of these patterns (e.g., [31]). Spatial distribution of polymorphism around the target of selection is often used to distinguish the footprints of demography and selection and to detect sweeps (e.g., [32]). The terminology describing distances and positions in the genome can be confusing, as one has to distinguish between physical (referring to sequence) and recombinational (referring to genetic map) positions. To avoid this confusion, hereafter we refer to the former as “genomic” and to the latter as “genetic” positions or distances.

Studying sweeps, especially adaptive introgressive sweeps, is useful for understanding evolutionary processes. Introgression may be a significant factor facilitating adaptation and speciation [33,34], yet the role of adaptive introgression in evolution is not fully understood [35]. De novo adaptive mutations do not arise frequently in small populations [33]; hence, introgression could be a crucial mechanism for species with small population size to gain adaptive potential. Systems used to study adaptive introgression should ideally be ones with hybrids of varying fitness so the adaptive traits can be identified easily, such as rapid adaptive radiations [35] in cichlids [36,37] and Darwin’s finches [38]. Other examples of adaptive introgression include the high-elevation *Senecio hercynicus* and low-elevation *Senecio ovatus* in Germany, where introgression from the former species aided the latter in adapting to climatic conditions at high elevations and shorter vegetative phases [39]. Serpentine adaptation in *Arabidopsis arenosa* was reported to occur due to introgression of adaptive alleles from a related species [40]. Adaptive introgression in crop species or wild crop relatives is also abundant, as seen in Atlantic salmon [41], and in rice and its wild progenitor [42].

The occurrence and distribution of selective sweeps, whether they involve introgression, and whether the introgression involved in selective sweeps is adaptive or not, have gained attention as well. Adaptative introgression can drive local adaptation to climate (e.g., [43,44]) and to high elevation, e.g., as reported for the EPAS1 gene in Tibetans [45]. In Acer, introgression from high-elevation *Acer morrisonense* to the low-elevation *Acer caudatifolium* was suggested to facilitate the postglacial upward range expansion of the latter [46]. Introgression from the high-elevation-adapted maize *Zea mays* subsp. *mexicana* contributed to adaptation to highlands in various maize varieties which are otherwise restricted to lowlands (e.g., [47,48]). In the Qinghai–Tibet Plateau, introgression from high-elevation *Cupressus* species has provided the adaptive potential for the low-elevation or -latitude *Cupressus duclouxiana* in cooler and drier habitats at higher elevations and latitudes [49]. In contrast, adaptative introgression was not found to play any role in altitude adaptation in eastern gorillas [50].

This study focuses on two narrow-range *Senecio* species native to Mount Etna, Sicily. Typical *S. aethnensis* Jan ex DC. occurs above 2000 m, whereas typical *S. chrysanthemifolius* Poir. occurs below 1000 m [14,51]. The two species form hybrids at intermediate elevations [52,53]. Hybrids were shown to have variable fitness in the greenhouse setting [54,55,56,57]. *S. aethnensis* and *S. chrysanthemifolius*’ habitats show contrasting environmental conditions, both elevation- and ecology-related, such as temperature, solar radiation, and water availability [54,58,59,60]. Their divergence was estimated to have occurred less than 200,000 years ago, coinciding with the rise of Mount Etna to elevations above 2000 m due to volcanic activity [61,62,63]. Both species are short-lived, obligately outcrossing perennials pollinated by generalist insects and bear wind-dispersed seeds; they can be distinguished by many morphological and physiological characters, such as degree of leaf dissection and colour, size of capitula and florets, and flowering time [14,53,59,64].

So far, selection studies on the high-elevation *S. aethnensis* and low-elevation *S. chrysanthemifolius* on Mount Etna have focused on particular traits and genetic loci underlying divergent selection [14,16]. Selection for ecologically relevant genetic variants, estimated from the cline analyses in the *Senecio* hybrid zone, is strong, with the selection coefficient ranging from 0.16 to 0.78 [16]. Such strong selection is necessary to maintain narrow clines in the face of geneflow [8,9]. However, the genome-wide patterns of selection and introgression (due to frequent hybridisation) are less studied in this system. As the elevational *Senecio* cline on Mount Etna is a relatively well-characterised ecotonal hybrid zone maintained by adaptation to the contrasting environmental conditions, this system is ideal for studying selection during ecological speciation. Here, we aim to investigate the contributions of introgressive and non-introgressive sweeps to adaptation. Introgressive sweeps are driven by an allele that introgressed from other species, whereas non-introgressive sweeps represent locally adaptive (species-specific) selective sweeps that do not show signs of introgression. Given the likely prevalence of local adaptation to high- and low-elevation conditions in this system, we hypothesised that species-specific, non-introgressive sweeps should predominate and introgressive sweeps would be rare. Even though local, non-introgressive sweeps predominate, contrary to that a priori expectation, we detected numerous cases of likely introgressive sweeps, suggesting that adaptation in *Senecio* populations is facilitated by sharing alleles between species. This is likely due to constraints of their relatively small populations which limit the availability of the “right” adaptive alleles; sharing such alleles between the species provides a way to speed up adaptation.

## 2. Materials and Methods

### 2.1. Filtering of Raw Reads, SNP Calling and Polymorphism Analyses

Leaves from six *S. aethnensis* and six *S. chrysanthemifolius* individuals (Additional File: Supplementary Table S1) were used. Plants were grown from seeds collected on Mount Etna, Sicily, in 2002–2003. *S. aethnensis* seeds were collected from one population at 2600 m above sea level (masl), and *S. chrysanthemifolius* seeds were collected from two populations at 755 and 852 masl. They are assumed to be pure species, as the plants were morphologically typical of either species and from the species’ typical range (above 2000 masl for *S. aethnensis* and below 1000 masl for *S. chrysanthemifolius*). The three sampled populations are 11 km, 19 km and 29 km apart using map distances that ignore the landscape and thus underestimate the distance on the mountain. These populations were used in previous studies of the same systems (e.g., [61]). DNA was extracted using the QIAGEN DNeasy Plant Mini Kit (QIAGEN, Hilden, Germany) following the manufacturer’s protocol, using young leaves of the plants. Samples were then sequenced at the NERC Environmental Omics Facility (Liverpool, UK). Sequencing libraries with an insert size of ~300 bp and ~500 bp were created by the sequencing facility, and subsequently, both types of libraries were sequenced for all samples using the Illumina 1.9 technology (Illumina, Inc., San Diego, CA, USA). Two insert sizes were used for reasons unrelated to the analyses in the current paper.

Before SNP calling, sequence reads were first trimmed and filtered using default options in trimmomatic v0.36 [65] to remove low-quality reads and adaptors. Filtered reads were then aligned to the indexed reference *S. squalidus* genome [66] using BWA mem v0.7.15 [67]. *S. squalidus* originated only ~350 years ago as a homoploid hybrid between *S. aethnensis* and six *S. chrysanthemifolius* [68], making its genome a suitable reference for SNP calling in *S. aethnensis* and *S. chrysanthemifolius*. It is the only species among the three with an available reference genome [66]. SAMtools v0.1.19 [69] was used to create and sort bam files. Duplicates were marked and removed using Picard v2.0.1 [70]. BCF files containing genotype likelihoods were obtained using the mpileup function in SAMtools v0.1.19. Using BCFtools v1.23.1 [71], SNPs were then called using the call function, filtered using the vcfutils.pl varFilter function within BCFtools v.1.23.1 and finally exported as a VCF file. Indels were removed using VCFtools v0.1.13 [72]. The resulting VCF file contained all SNP data used in subsequent analyses.

Using the SNP dataset, genetic polymorphisms were analysed and compared among synonymous and non-synonymous coding sites as well as non-coding sites in the two species. We used a non-overlapping sliding window (10 kb) approach for the following groups of polymorphisms: (1) Tajima’s D (a statistical test calculating the difference between the mean number of pairwise differences and the number of segregating sites) and fixation index F_ST_ (a measure of population differentiation that quantifies the genetic distance between subpopulations) using all sites were carried out using VCFtools; (2) D_xy_ (a measure of absolute genetic divergence that calculates the average number of pairwise nucleotide differences per site between two populations) was estimated using pixy [73], using the bcf file containing all variant and invariant sites (before the vcfutils.pl varFilter step); (3) nucleotide diversity (π) from synonymous, non-synonymous and non-coding sites, Tajima’s D from synonymous sites, and ZnS (an estimate of linkage disequilibrium that calculates the average squared correlation of allelic identity across all pairwise combinations of polymorphic sites within a genetic locus) from all sites were estimated using ProSeq4 [74], with positions with missing data included in the analyses; and (4) gene count was determined using the *S. squalidus* reference genome [66]. The Kruskal–Wallis (KW) test was carried out to test any significant differences between the two target species in (1) to (3). Pearson’s correlation between gene count (per 10 Mb) and the number of non-introgressive and introgressive sweeps in each species was further estimated (see below for sweep detection and classification).

**Figure 1:**
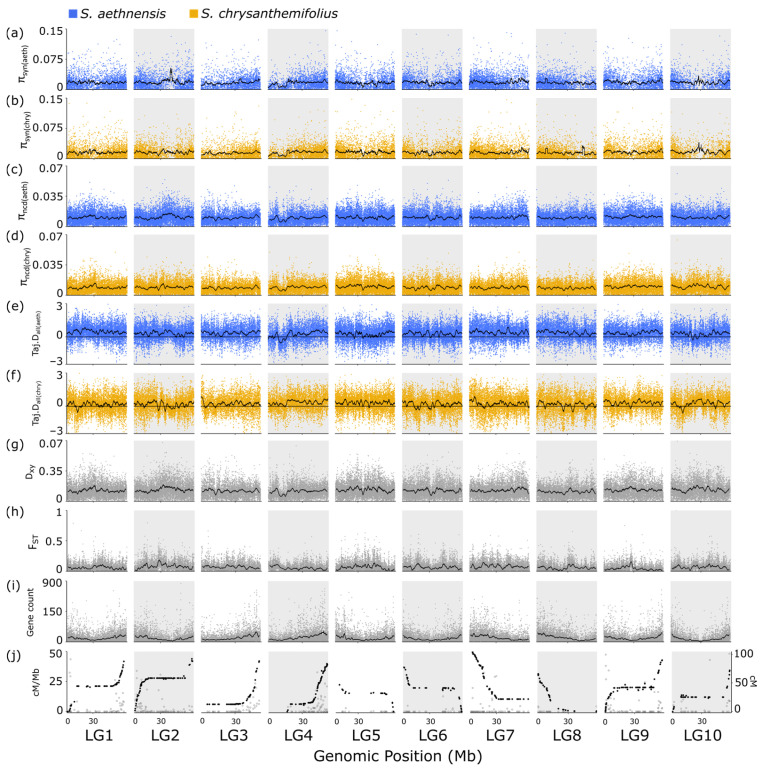
Genome-wide patterns for polymorphism and recombination summary statistics. Plots from top to bottom are (**a**,**b**) nucleotide diversity (π) (synonymous sites) for *S. aethnensis* and *S. chrysanthemifolius*, respectively; (**c**,**d**) nucleotide diversity (π) (non-synonymous sites) for each species; (**e**,**f**) Tajima’s D (all sites) for each species; (**g**) D_xy_ between the two species; (**h**) F_ST_ between the two species, with π, Tajima’s D and D_xy_ plots showing non-overlapping windows of 10 kb; (**i**) gene count (per 10 Mb); (**j**) estimated recombination rate (grey circles, left y-axis) and cumulative genetic distance (black circles, right y-axis) plotted against corresponding physical distance (X-axis), with outliers removed and with both using markers from [61]. The black lines in (**a**–**i**) denote the moving averages of every 200 windows (each 10 kb).

Genetic distances (cM) in the previously published genetic map [61] were used to estimate local recombination rates (cM/Mb). Genomic positions (Mb) of all the markers were identified (ones with exact matches and no duplicates) with blast-search against the *S. squalidus* reference genome [66] (*S. squalidus* is the ex situ homoploid hybrid species from the two target species in this study), after removing ambiguous matches. The local recombination rate (cM/Mb) was calculated by dividing the difference in linkage map locations (cM_2_–cM_1_) by the difference in genomic positions between each pair of markers closest to one another (Mb_2_–Mb_1_). Recombination rates over 50 cM/Mb were treated as errors and discarded. To have an overview of the effect of recombination rate on the size of sweep regions, the lengths of all overlapping windows that were outliers in RAiSD v2.8 [30] or VolcanoFinder v1.0 [75] analyses were compared between actively and rarely recombining regions. All overlapping sweep windows were grouped to a single sweep region in this analysis. In addition, using the genome and linkage map, nucleotide diversity (π), Tajima’s D and gene count between high- and low-recombining regions were compared across the genomes of *S. aethnensis* and *S. chrysanthemifolius*. High-recombining regions are defined as the regions where the cumulative recombination curve (shown in Figure 1j) increases or decreases with increasing position on the X-axis, whereas low-recombining regions are defined as the regions where the cumulative recombination curve is flat. All the above statistics were plotted against genomic positions using the R package ggplot2 [76].

### 2.2. Genomic Scans for Signals of Adaptive Introgression and Selective Sweeps

VolcanoFinder v1.0 [75], a tool specifically developed for detection of selective sweeps involved in adaptive introgression, was used to detect introgressive sweeps. It uses the patterns of excess intermediate-frequency polymorphism produced in the flanking region of the sweep, which results in a volcano shape in pairwise genetic diversity [75]. VCF files containing SNP information were first converted to allele frequency files using VCFtools v0.1.13 [72]. The resulting files were run through SweepFinder2 v1.0 [77] to generate site frequency spectrum (SFS) files. SweepFinder2 was then run, with spacing between grid points (-sg option) set to 1000. Composite likelihood ratio (CLR) peaks exceeding the 99th percentile were considered outliers in each linkage group. VolcanoFinder was then run using the same SFS file, with the test site (-ig option) set at every 1 kb and with model number 1. Composite likelihood ratio (CLR) peaks exceeding the 95th percentile were considered outliers in each linkage group. The power of VolcanoFinder to detect adaptive introgression sweeps is very similar to the power of SweepFinder2 to detect classic sweeps [75], perhaps because the former is a derivative of the latter and the two programs use the same type of data (allele frequency) and the same composite likelihood approach to detect sweeps.

RAiSD [30] was used to detect selective sweeps (that may or may not be involved in introgression). This software uses the μ statistic, which is a composite evaluation test that tracks fluctuations in the amount of polymorphism, level of LD and SFS along chromosomes [30]. Unlike VolcanoFinder, it does not explicitly search for signatures specific to introgression; hence, it does not have the ability to distinguish between introgressive and non-introgressive sweeps, as both types of sweeps show similar fluctuations in polymorphism LD and SFS. Sliding window size (-w; in terms of number of SNPs and not based on the genomic scaffolds) was set to 100, and the probability value (-A) was set to 0.995. μ values exceeding the 99th percentile of each linkage group were considered outliers.

To further analyse signals of sweeps, selscan [78] was used to calculate iHH12, a haplotype homozygosity-based statistic that is demonstrated to have reasonable power to detect both hard and soft sweeps [79]. The input vcf file containing phased data was generated using the vcf_phase.py script in the Python 3 package Pop-Gen Pipeline Platform (PPP) [80]. VCFtools (--plink flag) was used to generate the input mapping file. We used a default 5% cutoff for SNPs with minor allele frequency. Subsequently, we used the –norm flag in selscan to normalise iHH12 values for all LGs in each species. iHH12 values exceeding the 99th percentile were treated as outliers, and only outlier regions from VolcanoFinder or RAiSD analyses that contained iHH12 outliers were considered true sweeps. The number of SweepFinder2 outliers present in putative VolcanoFinder sweep regions was also counted.

Bayescan v2.1 [81], a F_ST_-based software, was run on all SNPs in order to identify the likely introgressed regions. Classic selective sweeps (within species) inflate F_ST_ locally, whereas introgressive sweeps should exhibit low F_ST_ near the target of selection due to sharing of alleles. SNP datasets of the two species were run in 80,000 SNP chunks that are randomly sampled and non-repeating to reduce computational load. SNPs were considered outliers if their respective log10(PO) > 1. The number of Bayescan outliers in each of the putative RAiSD and VolcanoFinder sweep regions was estimated.

In this study, we focus on two types of sweeps: (1) introgressive sweeps driven by an allele that introgressed from the other species, with all outlier sweeps detected by VolcanoFinder (designed to detect introgression) and those by RAiSD (does not differentiate different types of selective sweeps) that overlap with VolcanoFinder outlier regions (>10 kb) considered introgressive, and (2) local (species-specific), non-introgressive sweeps where no signs of introgression are detected. All outlier sweeps detected by RAiSD that do not overlap with those from VolcanoFinder are considered non-introgressive. A paired *t*-test was carried out to test the differences between the number of introgressive and non-introgressive sweeps, using the stats package included in R.

### 2.3. Gene Ontology (GO) Enrichment Analysis

GO enrichment analysis was carried for introgressive and non-introgressive sweep regions in each species using the Blast2GO pipeline [82] that is implemented in the OmicsBox v3.0 software [83] using the default settings of the Fisher’s exact test option (FDR value < 0.05). Annotations of the *S. squalidus* reference genome [66] were used in all enrichment analyses.

### 2.4. Selection Coefficient and Age of Sweeps

The selection coefficient (s) was estimated using s = r*ln(2N_e_)/α (see theoretical explanations in [29,84]), where r = recombination rate, N_e_ = effective population size, and α = sweep strength obtained from VolcanoFinder. The age of sweeps (T) was estimated using T = 2*In(2N_e_)/s [85], where N_e_ = effective population size and s = selection coefficient.

The recombination rate (cM/b) was obtained by first fitting a generalised additive model (GAM) to each LG using the mgcv::gam function in the R package modelbased [86], then estimating the derivative of the fitted curve using the estimate_slope function, divided by 100 (as 1 cM corresponds to 1% chance of crossovers). Subsequently, r of a certain outlier sweep region was estimated by averaging the r at the first and last window of the region. Effective population sizes estimated in [87] were used (*S. aethnensis*: 16,482; *S. chrysanthemifolius*: 11,398; both lower estimates of the size range reported). As only VolcanoFinder gives the α statistics, only sweeps from this analysis were used for the estimation of the selection coefficient and age of sweeps.

### 2.5. Matching Previously Identified Highly Differentiated SNPs with Outliers in This Study

The 76 previously identified highly differentiated loci (151 bp-long markers) from [16] were blast-searched against the *S. squalidus* reference genome to examine whether they lay in any outlier introgressive or non-introgressive sweep regions identified by VolcanoFinder and RAiSD. In the case of more than one match, the one with at least a 95% identity match and a length of 140 bp was chosen. Markers with matches on multiple scaffolds and identical match statistics were disregarded. For markers with multiple matches on the same scaffold, the match with no gaps and a higher percentage match was chosen. Regardless of whether the 76 loci lay in outlier sweeps or not, their selection coefficients were estimated using (1) the cline width estimated in [16], by s = 3(σ/w)^2^, where σ denotes a dispersal rate of 323 m/gen (higher estimate in [16]) and w denotes cline width (m) [8,88,89], and (2) the α statistic estimated in VolcanoFinder for the corresponding position in this study. Pearson’s correlation between the selection coefficients of these loci in the following categories was also estimated (1) between estimates from using the α (from VolcanoFinder) of *S. aethnensis* and *S. chrysanthemifolius* in this study, (2) between estimates from using the α of *S. aethnensis* in this study and that from the results in reference [16], and (3) between estimates from using the α of *S. chrysanthemifolius* in this study and that from the results in reference [16].

## 3. Results

To collect genome-wide DNA polymorphism data, we re-sequenced the genomes of six *S. aethnensis* and six *S. chrysanthemifolius* accessions (Additional File: Supplementary Table S1). While the sample of 12 individuals may seem small, it has been theoretically shown to be sufficient for coalescent-based population genetic inference, with larger samples leading to diminishing returns [90,91]. The reads from sequenced individuals were mapped to the *Senecio squalidus* reference genome sequence [66] and SNPs called and filtered as described in the Materials and Methods section. After SNP filtering and after all indels were removed, a total of 22,461,434 SNPs in 10 linkage groups (LGs) across both species were used for analyses (Table 1). The number of SNPs ranged from 1,742,806 to 2,398,778 SNPs per LG, and SNP density per scaffold ranged from 0.033 to 0.038 SNPs per base in the sample including both species (Table 1).

### 3.1. Polymorphism Across the S. aethnensis and S. chrysanthemifolius Genomes

The genome-wide distributions of the statistics summarising DNA polymorphisms and species divergence (Tajima’s D, π, D_xy_ and F_ST_) are plotted in Figure 1 and in Supplementary Figure S1. The genetic diversity statistics described below are listed in Additional File: Supplementary Table S2, and the results of the Kruskal–Wallis (KW) tests comparing various polymorphism statistics between *S. aethnensis* and *S. chrysanthemifolius* can be found in Additional File: Supplementary Table S3.

Mean nucleotide diversity (π) across the genome was higher in *S. aethnensis* than *S. chrysanthemifolius* in all three site categories (synonymous sites π_syn_: 0.0176 ± 0.0214 versus 0.0155 ± 0.0198; non-synonymous sites π_nsyn_: 0.0112 ± 0.0103 versus 0.0098 ± 0.0097; non-coding sites π_ncd_: 0.0103 ± 0.0062 versus 0.0091 ± 0.0057 for *S. aethnensis* and *S. chrysanthemifolius*, respectively). Mean nucleotide diversity was significantly higher in *S. aethnensis* than that in *S. chrysanthemifolius* regardless of the site category across all individual LGs, except πsyn in LG 5 (*p*-value = 0.053). Across the genome of each species, frequently recombining regions had significantly higher π than rarely recombining regions in all three site categories (Supplementary Figure S2).

Mean Tajima’s D across the genome was also higher in *S. aethnensis* than *S. chrysanthemifolius* in both site categories analysed (all sites Taj.D_all_: 0.3099 ± 0.5960 versus 0.2109 ± 0.6767; synonymous sites Taj.D_syn_: 0.0784 ± 0.8778 versus −0.0290 ± 0.9178 for *S. aethnensis* and *S. chrysanthemifolius*, respectively). All comparisons for Taj.D were significant between the two species except LG 5 (KW test χ^2^ = 0.026, *p*-value = 0.87) and LG 9 (KW test χ^2^ = 3.49, *p*-value = 0.062) for synonymous sites and LG 10 (KW test χ^2^ = 0.88, *p*-value = 0.35) for all sites. Tajima’s D of *S. aethnensis* was higher than that in *S. chrysanthemifolius* for both site categories in all LGs except for Taj.D_all_ in LG 4 and 5, and for Taj.D_syn_ in LG 4 (although differences were insignificant for LG5, 9 and 10 for the site categories mentioned above). Taj.D_syn_ was >0 for all LGs in *S. aethnensis*, whereas it was less than 0 for all LGs except LG 4 in *S. chrysanthemifolius*. Across the genome of each species, frequently recombining regions had significantly higher Tajima’s D than rarely recombining regions in all three site categories (Supplementary Figure S2).

Mean ZnS for *S. aethnensis* and *S. chrysanthemifolius* was 0.2235 ± 0.0800 and 0.2506 ± 0.1017, respectively. ZnS of *S. aethnensis* was significantly smaller than that in *S. chrysanthemifolius* in all LGs, reflecting less linkage disequilibrium across the genome of *S. aethnensis* compared to *S. chrysanthemifolius*.

Mean D_xy_ and F_ST_ between the two species across the genome were 0.0125 ± 0.0074 and 0.0773 ± 0.0640, respectively, indicating relatively low genetic differentiation between the two species. Chromosomal scaffolds corresponding to linkage groups (LG) 1, 2, 5, 6, 9, 10 had rarely recombining regions in the middle of the LGs, with recombination occurring primarily at the ends of the LGs, whereas LG 3, 4, 7, 8 actively recombined on one side and contained rarely recombining regions on the opposite side (Figure 1j). On all chromosomes, the rarely recombining regions showed slightly less diversity within species (π, Figure 1a,b and Supplementary Figure S1a,b) and somewhat higher divergence between species (D_xy_, Figure 1g; Fst, Figure 1h) compared to more actively recombining regions.

Gene count (per 10 Mb) was significantly higher in frequently recombining regions than that in rarely recombining regions (*p*-value < 2.2 × 10^−16^).

### 3.2. Genome Scans for Sweep Signals

**Figure 2:**
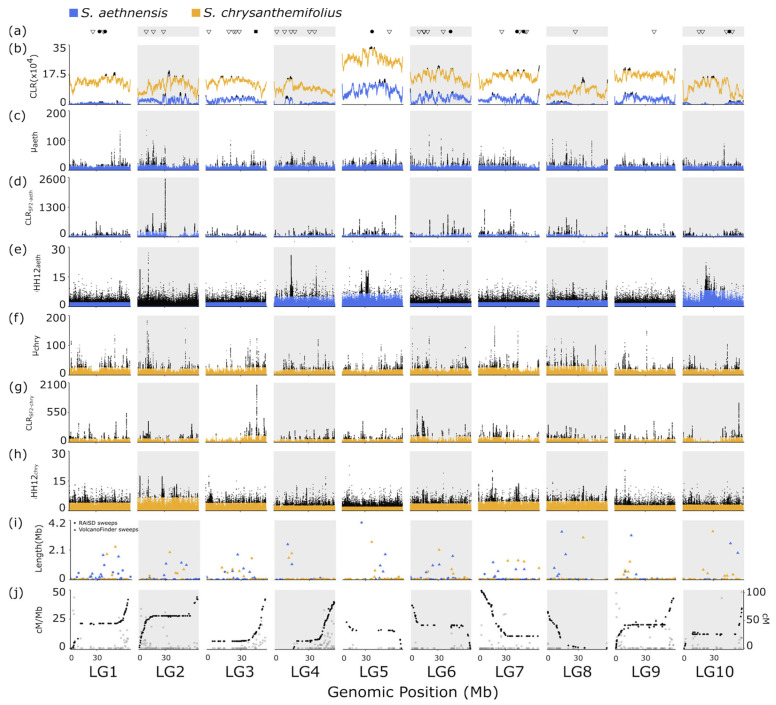
Genome-wide scans for selective sweeps. Plots from top to bottom are (**a**) position of loci under selection identified in cline-based analysis in [16]. Black filled circles and black filled squares are outliers that are located within outlier adaptive introgressive sweeps detected by VolcanoFinder and within non-introgressive sweeps detected by RAiSD, respectively, in either or both species. Open triangles are ones that are not found within sweep regions detected in this study. (**b**) Composite likelihood ratio (CLR) from the scans for adaptive introgression sweeps using VolcanoFinder. test sites were set to be every 1 kb, with the top 5% of data points in each LG treated as outliers (black filled circles); (**c**,**f**) µ statistic from the scans for positive sweeps using RAiSD for *S. aethnensis* and *S. chrysanthemifolius*, respectively. Sliding window size (in terms of number of SNPs) is 100, with the top 1% of data points in each LG treated as outliers (black filled circles). (**d**,**g**) CLR from SweepFinder2. Test sites were set to be every 1 kb, with the top 1% of data points in each LG treated as outliers (black filled circles). (**e**,**h**) Normalised iHH12 values estimated using selscan (default parameters) for each species. (**i**) Distribution of length of sweeps (total length of consecutive windows that are outliers in RAiSD and VolcanoFinder analyses), plotted at the mid-point of each sweep region (same as in Figure 1i). (**j**) Estimated recombination rate (grey open circles, left y-axis), and cumulative genetic distance and corresponding physical distance with outliers removed (black filled circles, right y-axis), both using markers from [61]. Panel (**h**) is the same figure as Figure 1j. They were repeated as they are useful to compare with other panels in both figures.

The results of the selection scans are shown in Figure 2. Only the outlier sweep regions (in RAiSD or VolcanoFinder) that were also iHH12 outliers were considered true outlier sweeps in this study. This is a widely used conservative approach (e.g., [92,93]) to exclude false positive signals, as different methods have different biases and intersection of their outliers is more likely to contain true sweeps, though at the cost of losing a fraction of true positives that were detected only by one of the methods. The details of the outlier sweeps can be found in Supplementary Tables S4 and S5.

#### 3.2.1. Non-Introgressive Versus Introgressive Sweeps

**Figure 3:**
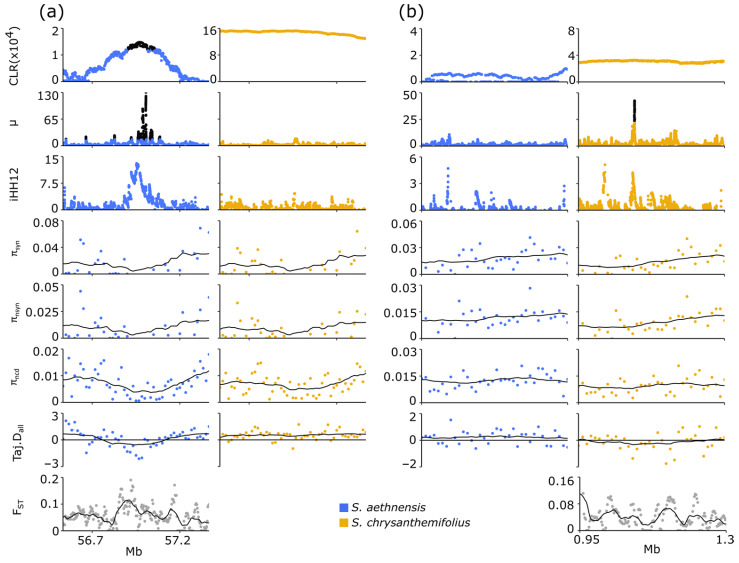
Examples of likely introgressive and non-introgressive types of sweeps. Blue plots are for *S. aethnensis* and yellow plots are for *S. chrysanthemifolius*. The black data points in the top two panels are outlier sweep regions detected by VolcanoFinder and RAiSD, respectively. The black lines denote moving averages. The no. of windows for estimating moving averages for (**a**,**b**) is 20 and 10, respectively (based on length of sweep regions presented). (**a)** Introgressive sweep region in LG 1, with overlapping outlier sweep regions from VolcanoFinder and RAiSD for *S. aethnensis* only; (**b**) non-introgressive sweep region for *S. chrysanthemifolius* only in LG 2. Both sweep regions are supported by a peak in normalised iHH12 values, a drop in π (all types of sites), a negative Tajima’s D and a peak in F_ST_.

**Figure 4:**
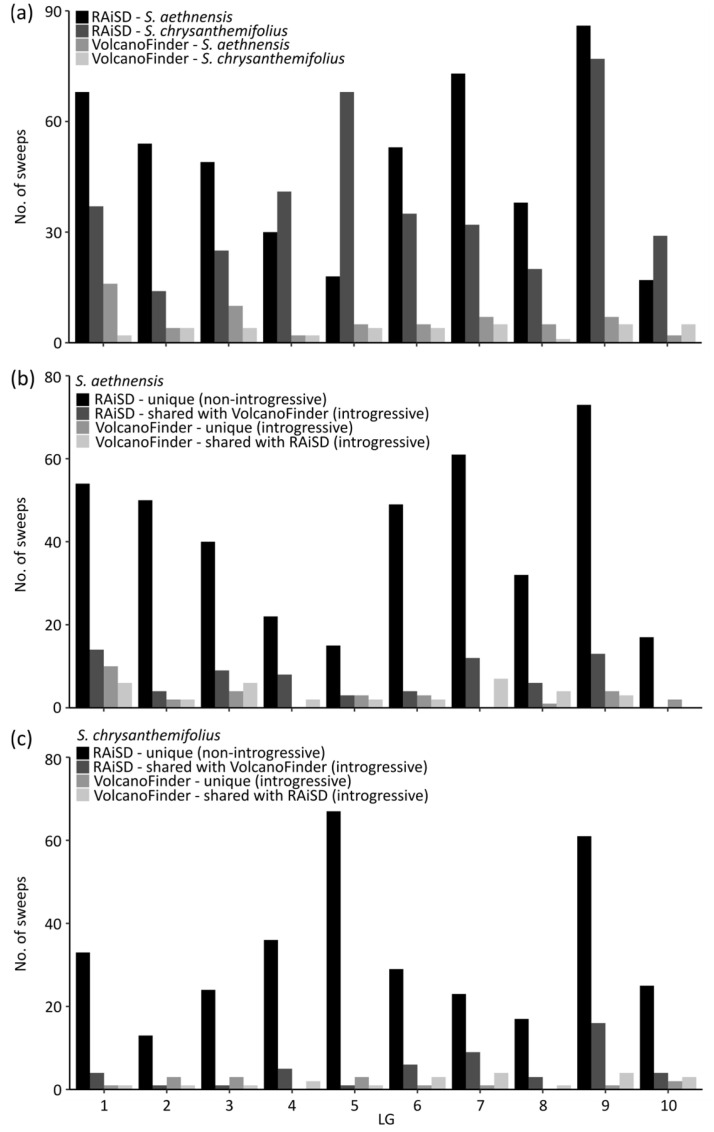
Comparison of number of sweep regions in *S. aethnensis* and *S. chrysanthemifolius* using VolcanoFinder and RAiSD. (**a**) Total number of sweep regions in each species detected by each software; (**b**) number of sweep regions in *S. aethnensis*; (**c**) number of sweep regions in *S. chrysanthemifolius*. For each LG in (**b**,**c**), bars from left to right denote unique RAiSD sweep regions (non-introgressive), RAiSD sweep regions that overlap with VolcanoFinder’s (introgressive), unique VolcanoFinder sweep regions (introgressive), and VolcanoFinder sweep regions that overlap with RAiSD’s (introgressive), respectively.

An example of each of the two types of sweeps (introgressive and non-introgressive) is presented in Figure 3. All types of sweeps were supported by peaks in iHH12, low π, low Tajima’s D, and a peak in F_ST_. Overall, genomic regions affected by selective sweeps were longer (in terms of physical size) in regions of low recombination (Figure 2i,j). In both species, sweep regions detected by RAiSD were mostly non-introgressive, with a small number of sweep regions overlapping with those identified by VolcanoFinder (thus classified as introgressive sweep regions) (Figure 4b,c). Although SweepFinder2 outliers were present in some VolcanoFinder putative sweep regions (Additional File: Supplementary Table S5), they corresponded to only a small part of the introgressive regions.

**Figure 5:**
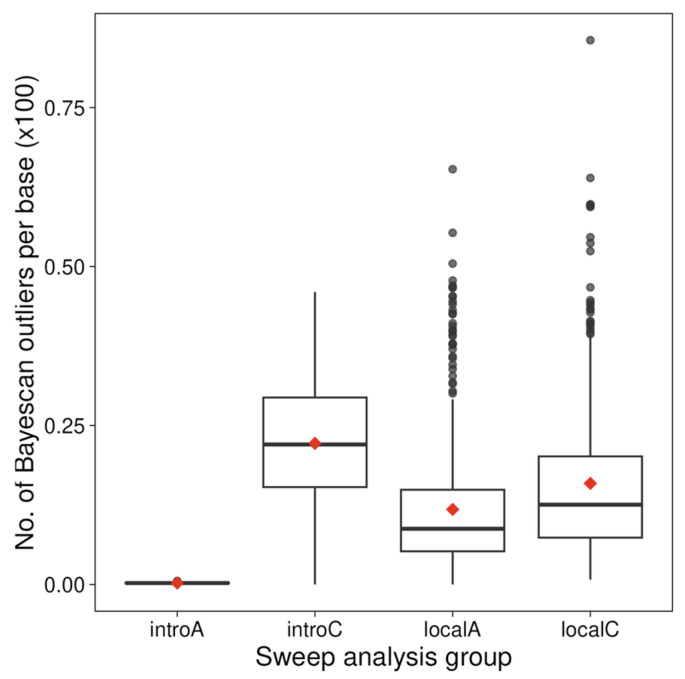
Boxplots comparing the number of Bayescan outliers (per base) in putative sweep regions for each species. Red data points denote the mean number of outliers. Abbreviations for sweep analysis group on the X-axis: intro = introgressive sweeps, local = locally adaptive sweeps, A = *S. aethnensis*, C = *S. chrysanthemifolius*.

All Bayescan outliers had F_ST_ > 0.75. Introgressive sweeps in *S. aethnensis* showed the fewest Bayescan outliers (close to none) (Figure 5). Introgressive sweeps in *S. chrysanthemifolius* contained the highest number of outliers among the four sweep groups, higher than that in locally adaptive sweeps in both species (Figure 5).

The paired *t*-test showed that the number of non-introgressive sweeps in each species was significantly higher than that of introgressive sweeps (*S. aethnensis*: t = −6.20, *p*-value = 0.0002; *S. chrysanthemifolius*: t = −5.25; *p*-value = 0.0005), whereas the numbers of either introgressive or non-introgressive sweeps between the two species were not significantly different (introgressive: t = 1.84, *p*-value = 0.10; non-introgressive: t = 1.00, *p*-value = 0.34).

In LG 10 of *S. aethnensis*, none of the sweeps were shared between RAiSD and VolcanoFinder (Figure 4b). Regarding sweep regions detected by VolcanoFinder (all introgressive), all regions overlapped with those detected by RAiSD in LG 4 for both species, LG 7 for *S. aethnensis* and LG 8 for *S. chrysanthemifolius*. For other LGs (except for LG 10 of *S. aethnensis*, described above), there were more unique than shared VolcanoFinder sweep regions in four and three LGs for *S. aethnensis* (LG 1, 5, 6, 9) and *S. chrysanthemifolius* (LG 2, 3, 5), respectively, whereas there were more shared VolcanoFinder regions than unique ones for two and four LGs for *S. aethnensis* (LG 3, 8) and *S. chrysanthemifolius* (LG 6, 7, 8, 10), respectively.

#### 3.2.2. Correlation Between Sweep Number and Gene Density

The numbers of sweeps detected were variable across the entire genome. RAiSD detected more outlier sweep regions than VolcanoFinder in each species (486 and 63, respectively, for *S. aethnensis*; 378 and 36, respectively, for *S. chrysanthemifolius*; all supported by iHH12 outliers) (Figure 4, Additional File: Supplementary Table S6). There were also more sweeps detected in *S. aethnensis* than in *S. chrysanthemifolius* for both RAiSD and VolcanoFinder, except for RAiSD in LG 4, 5 and 10, as well as VolcanoFinder in LG 10 (Figure 4a).

Pearson’s correlation tests showed that the number of non-introgressive sweeps had significant positive correlation with gene count for both species (*p*-value = 0.0001 and 0.0011 respectively) (Supplementary Figure S3). For introgressive sweeps, *S. aethnensis* did not show a significant correlation with gene count (*p*-value = 0.4724), whereas *S. chrysanthemifolius* showed a significant negative correlation with gene count (*p*-value = 0.0498) (Supplementary Figure S3).

#### 3.2.3. Strength of Sweep Signals

LG 5 showed stronger introgressive signals than the rest of linkage groups for both species: it was the only LG with CLR scores over 250,000 and 100,000 for *S. chrysanthemifolius* and *S. aethnensis*, respectively. In LGs 5, 6, 7, and 9, distributions of CLR scores had similar patterns in both species. In VolcanoFinder and RAiSD analyses, strong sweep signals were detected throughout the linkage groups and not concentrated in the particular regions. Similarly, outliers were observed throughout the linkage groups in the iHH12 analyses. There were strong iHH12 signals observed in LG 2, 4, 5 and 10 for *S. aethnensis* and LG 2, 3, 7 and 9 for *S. chrysanthemifolius* (Figure 2, Supplementary Figures S1 and S4). In LG2, there was a stretch of low Tajima’s D between 35 and 50 Mb (Supplementary Figures S1 and S4) that also corresponded to very high iHH12 values in *S. chrysanthemifolius*. There were also increased signals for adaptive introgression at the ends of each LG, as shown in the CLR plots for VolcanoFinder (Supplementary Figures S1 and S4).

### 3.3. Recombination Rate, Selection Coefficient and Age of Sweeps

Strength of selection can be quantified with the selection coefficient (s) calculated from α estimated by VolcanoFinder: s = r×ln(2N_e_)/α [29,84], where r is local recombination rate and N_e_ is effective population size. We used N_e_ estimates from [87] (*S. aethnensis*: 16,482; *S. chrysanthemifolius*: 11,398). Local recombination rates, inferred with GAM (see Materials and Methods), varied across the genome, with the highest recombination rate in each LG ranging from 4 × 10^−6^ to 11 × 10^−6^ cM/b (Supplementary Figure S5). Using these estimates of local recombination rate, the estimated selection coefficients ranged from 0.00 to 0.98 in *S. aethnensis* (after disregarding one sweep with s > 1) and from 0.00 to 0.76 in *S. chrysanthemifolius*.

**Figure 6:**
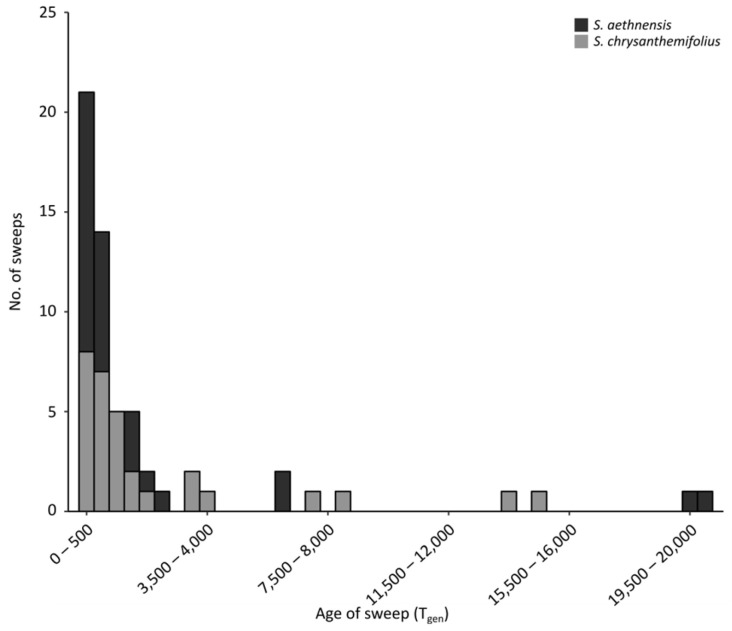
Distribution of the age of sweeps identified by VolcanoFinder in this study. Sweeps with an age of more than 20 ky are excluded (details in Additional File: Supplementary Table S5).

The inferred age of sweeps ranged from 18 to 81,904 generations in *S. aethnensis* (Additional File: Supplementary Table S5), after disregarding one sweep with an age estimate larger than 3 million generations, which exceeded the time of divergence between the two species < 200 kya [62]. The age of sweeps in *S. chrysanthemifolius* ranged from 27 to 161,915 generations (Figure 6; Additional File: Supplementary Table S5). There were two *S. chrysanthemifolius* sweeps with an age around 14 kya, which coincided with the time of rapid climate change around the last deglaciation event (Figure 6). We could not estimate r, s or sweep age for ten candidate outlier sweep regions (Additional File: Supplementary Table S5) located at the beginning or end of LGs, where the derivative for the fitted curve of the GAM could not be obtained.

**Figure 7:**
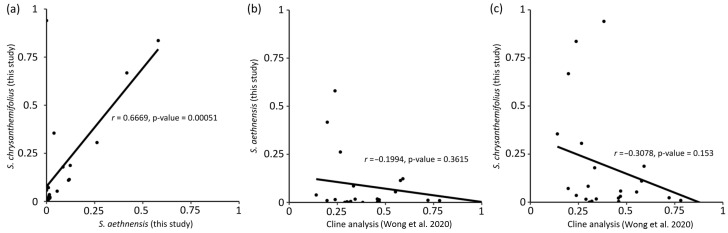
Comparisons of selection coefficients from the same genomic regions estimated in this study and [16]. The estimates in this study were calculated from α (VolcanoFinder), while in [16], selection coefficients were estimated from cline analysis. The comparisons are between (**a**) *S. aethnensis* and *S. chrysanthemifolius* estimates from α (VolcanoFinder) in this study, (**b**) between *S. aethnensis* estimates in the two studies, and (**c**) between *S. chrysanthemifolius* estimates in the two studies.

Out of the 76 loci under strong selection identified using cline analysis in the previous study [16], 40 loci corresponded to the sweeps outlined above. For these 40 loci, the strength of selection could be estimated (and compared) by two independent approaches—from alpha in the VolcanoFinder output (see above) and from the width of the cline, as carried out in [16]. However, 12 of these loci had to be excluded from analysis due to extremely narrow estimated cline width (<100 m) resulting in an estimated selection coefficient (s) over 1. Three more loci were discarded due to inability to obtain a recombination rate from the GAM of their respective LG. Selection coefficients of the remaining 25 loci were compared between estimations in [16] and from introgression scans in this study (Additional File: Supplementary Table S9) (the two studies used the same formula for estimations). The correlation between s estimates obtained by independent approaches in [16] and by this study were non-significant (Figure 7b,c), which is hardly surprising, because the VolcanoFinder estimates were based on a large genomic regions analysed in a few “pure” individuals from the top or bottom of the mountain, while the estimates from the cline were based on a single SNP analysed in a much larger sample including pure *S. aethnensis* and *S. chrysanthemifolius* as well as hybrids from intermediate altitudes. On the other hand, the correlation between the species using the same approach was strongly significantly positive (r = 0.6669, *p*-value = 0.00051), with a tendency for *S. chrysanthemifolius* to have somewhat higher s than *S. aethnensis* (Figure 7a).

### 3.4. GO Enrichment in Introgressive Sweeps in S. chrysanthemifolius

In total, *S. aethnensis* and *S. chrysanthemifolius* harboured 1056 and 721 putative genes in the regions affected by local sweeps, respectively, and 1951 and 951 genes in their introgressive sweeps, respectively. GO enrichment analyses yielded no over- or under-represented GO terms in the high-elevation *S. aethnensis*, regardless of the type of sweep. There was also no over- or under-represented GO term in non-introgressive sweeps (sweeps unique to RAiSD) in the low-elevation *S. chrysanthemifolius*. Only over-represented GO terms were present among the introgressive sweeps in *S. chrysanthemifolius* (Additional File: Supplementary Tables S7 and S8, Supplementary Figures S5–S8). Among these over-represented GO terms were those related to xenobiotics (GO:0042908, GO:0042910), vitamin metabolism (such as folic acid and pyridine) (e.g., GO:0019363, GO:0072525, GO:0019362) and alkaloids (such as pteridine) (e.g., GO:0046655, GO:0046656; GO:0042559) (Additional File: Supplementary Table S8, Supplementary Figures S5–S8).

## 4. Discussion

This study analysed the signatures of selective sweeps throughout the genomes of hybridising *S. aethnensis* and *S. chrysanthemifolius* inhabiting high- and low-elevations, respectively, on Mount Etna, Sicily. The detected selective sweeps appeared to be mostly non-introgressive (but see caveats below) and widespread throughout the genome. Gene ontology enrichment analysis revealed several over-represented GO terms in introgressive sweeps in *S. chrysanthemifolius* that may be related to survival on Mount Etna (but no over- or under-represented GO terms in other types of sweeps or species). Two identified sweeps have ages close to the time of the last deglaciation event (~14 kya), and one other has an age close to the estimated divergence time of the two target species, suggesting that these events could impose selective pressures that are strong enough to still be detectable nowadays despite their signals fading over time. However, these three sweeps only accounted for a minor proportion of all selective events in the genomes of the two species and thus may not be representative.

### 4.1. Genetic Diversity Between Frequently and Rarely Recombining Regions

Although it was not apparent in genome-wide plots, separate analysis comparing genetic diversity between frequently and rarely recombining regions revealed that both π and Tajima’s D were significantly higher in frequently-recombining regions for all three site categories analysed. Similarly, gene density was significantly higher in frequently recombining regions. This fits theoretical expectations and empirical observations in other species that genetic diversity is higher in frequently recombining regions due to weaker linkage disequilibrium there (e.g., [5,94,95,96,97,98]).

*S. chrysanthemifolius* showed negative mean Tajima’s D_syn_ in nine out of ten LGs (compared to only one LG in *S. aethnensis*). This could be due to recent population expansion (e.g., post-glacial) as demonstrated in demographic models [16,87]. Another potential explanation is that *S. chrysanthemifolius* experienced more recent sweeps than *S. aethnensis* (the oldest sweeps tested in this study occurred in *S. aethnensis*). These sweeps could be driven by climate change—recent, hotter and dryer conditions at low elevation on Mount Etna are far from the environmental conditions experienced by its ancestors, leading to stronger sweep signals and thus lower Tajima’s D.

### 4.2. The Landscape of Selective Sweeps in Senecio

Divergent selection is key to maintaining species divergence between *S. aethnensis* and *S. chrysanthemifolius* (e.g., [52,56,66,99]). In contrast to previous *Senecio* studies that detected loci under selection based on changes in allele frequency and trait values along elevational clines (e.g., [14,16]), this study applied alternative approaches through genomic scans that are based on footprints that adaptation leaves in the site frequency spectrum, linkage disequilibrium and haplotype homozygosity. Comparing introgressive versus non-introgressive sweeps, we found that most sweeps were non-introgressive (in terms of number of sweep regions), which may, at least partly, be due to differences in power to detect these types of sweeps. However, the power of VolcanoFinder to detect adaptive introgression sweeps is very similar to the power of SweepFinder2 to detect classic sweeps [75], suggesting that introgressive sweeps are genuinely less common than classic intra-species sweeps in *Senecio*. Nevertheless, outlier introgressive sweeps were detected in every LG in both species. A potential explanation is that the prevalence of local sweeps reflects adaptation to contrasting environments of high and low altitudes on Mount Etna, while introgressive sweeps reflect adaptation to shared selective factors, such as parasites and infections spreading across both species.

We detected numerous sweep regions in genes likely involved in local adaption. It may be assumed that species adapted to contrasting environments evolve under strong local adaption, with introgression being maladaptive, as it brings genetic variants from a species adapted to different conditions [100,101,102]. However, the numerous putative adaptive introgressions identified in this study suggest that adaptive introgression is likely common between diverging species adapting to contrasting environments. The environment currently inhabited by *S. chrysanthemifolius* likely differs from its ancestral environment. Thus, the spread of alleles beneficial only for common features of both habitats, such as volcanic substrates and defence responses (discussed further below), may be causing adaptive introgressions. This scenario of infrequent gene flow coupled with few adaptive introgressive alleles is similar to other studies in *Senecio* [54] and other systems (e.g., [103,104]) in which regions involved in reproductive isolation are often resistant to introgression, while other genomic regions introgress.

### 4.3. Distribution of Selective Sweeps Along the LGs

A fairly sharp increase in signals for adaptive introgression was often observed towards the ends of the LGs, which had a higher recombination rate than the middle of the LGs. This could suggest a positive relationship between recombination rate and level of introgression. More frequent recombination at the distal ends of chromosomes, compared to the pericentromeric region, is well documented in plant species (e.g., [57,98,105,106,107,108]). Such actively recombining regions are typically gene-rich (e.g., [109,110]), hence possessing a higher chance for accumulating adaptive alleles. Higher gene density in the actively recombining regions (as shown in this and other studies) may, at least partly, account for more frequent selective sweeps in these regions. However, this positive correlation between gene density and abundance of sweeps (as well as between recombination rate and abundance of sweeps) is only shown for non-introgressive sweeps in this study. Such correlation is not observed for introgressive sweeps, possibly due to the small sample size of introgressive sweeps (up to 6) in each 10 Mb region. Regardless of the presence/absence of correlation between gene density and sweep abundance, both types of sweeps affected wider regions at rare recombination. This can likely be attributed to more extensive linkage disequilibrium in these regions (e.g., [5,95,96,97,111]). However, this should be interpreted with caution, as it may also be due to different sensitivities of selection tests in different regions of the chromosome due to differences in local linkage disequilibrium (or recombination rate) and level of polymorphism.

### 4.4. Impacts of Adaptive Introgression on Survival at Different Elevations

Studies have shown the importance of adaptive introgression between populations from different environmental habitats (such as different elevations and latitudes) to aid in their adaptation to new environments (including adaptive radiations) in both plants (e.g., maize [47,48]; maples: [46]; cypress [49]; oak [44]) and animals (e.g., humans [45]; various vertebrates [112]; rodents [113]; Bos species complex [114]; adaptive radiation in fish [115]).

Only introgressive sweeps in *S. chrysanthemifolius* harboured over-represented GO terms in the enrichment analysis. Among these over-represented GO terms were those that are likely extremely important for survival on Mount Etna. For instance, pyridines have crucial roles in defence against pathogens and herbivores (e.g., [116,117]). It has been observed that *S. chrysanthemifolius* close to the top limit of its range is susceptible to rust, whereas *S. aethnensis* does not appear to be as susceptible (authors’ observations). A well-known member of this group of alkaloids is nicotine, which is involved in defence responses in *Nicotiana* species, including the tobacco plant (e.g., [118,119]). Various GO terms associated with folic acid and its major constituent pteridine were also over-represented. They have been linked to increased oxidation and UV-B exposure in peach fruits [120], an increase in antioxidant capacity [121,122], and tolerance to oxidative damage from salt stress in snap beans [123]. Last but not least, it is unsurprising that GO terms related to xenobiotics were over-represented, as volcanoes are a common source of xenobiotic compounds [124]. Similarly, both UV- and defence-related GO terms were identified as key traits in the introgression from wild, high-elevation mexicana maize into domesticated maize [48]. *S. aethnensis* samples used in this study were collected close to the lower range limit of the species, where they potentially experience less strong selection than ones close to the higher range limit. Together with hypothetical genome annotations, these could potentially explain the lack of over- or under-represented GO terms in the species.

Given that *S. aethnensis* has a relatively restricted habitat at high elevation on Mount Etna, the higher effective population size, higher frequency of selective sweeps (both introgressive and non-introgressive), and higher genetic diversity are intriguing. Given that the cool climate at the time of species divergence around 150 kya [63] resembled that of the current range of *S. aethnensis*, it is tempting to speculate that the current *S. aethnensis* population is a refugium of a much larger ancestral population (shown in demographic models [16,87]) inhabiting low elevations when the climate was cooler (i.e., during ice ages). The hotter climate of the current interglacial may therefore have forced this cold-adapted species to shift its habitat to high elevation on Mt. Etna, which restricted it to a relatively small population size, while the diversity in this species still corresponds to the large ancestral population. However, given morphological plasticity in *S. aethnensis* is non-adaptive outside of its native elevations (while that of *S. chrysanthemifolius* is) [59], it would require other ways of adapting to the new conditions during range shift to higher elevations (such as high UV and volcanic substrates). This explains the presence of numerous locally adaptive (non-introgressive) sweeps in this species.

### 4.5. Caveats

This study is based on the analysis of sequenced genomes of 12 individuals, which may seem a small sample size by modern standards. The question of optimal sample size has been thoroughly investigated in theoretical studies [90,91], which demonstrated that 12 is in fact the optimal sample size for coalescent-based population genetic inference, with larger samples leading to diminishing returns. This conclusion is rooted in population genetic theory and remains relevant for modern population genetic studies that often include much larger sample sizes. More recent theoretical analyses (e.g., [125]) are consistent with the earlier conclusion that a modest sample size of ~10 provides most information about the population, with larger samples leading to only modest gains. Different parts of a genome have different histories, which effectively samples the past history of a species. In fact, quite a lot of information can be obtained from a sample size of one individual, as illustrated by frequently used approaches to reconstruct past demography of a species from a single genome sequence (e.g., [126,127]).

One major caveat in interpreting selection scan results is the high rate of false positives, as it is difficult to simulate a biologically accurate model, which is confounded by numerous factors, including time, strength and density of selective sweeps, demography, recombination, mating system and so on [31,128]. Population size change also poses a challenge in sweep detection. This could bias our interpretation of introgressive and non-introgressive sweeps. However, VolcanoFinder, SweepFinder2 and RAiSD, used in this study, are moderately robust to population bottlenecks, except for scenarios with extreme bottlenecks in RAiSD [30,75]. Both VolcanoFinder [75] and SweepFinder2 [77] use the background (genomewide) site frequency spectrum as a reference, which corrects for past demographic history and makes these software tools robust to past changes in population size. VolcanoFinder is a derivative of SweepFinder, and they share the same composite likelihood-based approach and part of the code. Power analyses of these approaches to detect introgressive and non-introgressive sweeps were completed previously [75,92], making comparisons of the results of these two analyses more straightforward than the comparisons with unrelated approaches such as RAiSD and iHH12. In particular, Setter et al. [75] concluded that “for both strong and weak selection, the power of SweepFinder2 to detect classic sweeps closely corresponds to the power of VolcanoFinder to detect adaptive introgression sweeps”. Thus, the results of these two programs are directly comparable, and the larger number of sweeps detected by SweepFinder2 compared to VolcanoFinder sweeps indicates the prevalence of non-introgressive sweeps, though introgressive sweeps are also not uncommon in the two *Senecio* species on Mount Etna.

Sweep detection is further complicated by the fact that one cannot make inferences about selection and recombination, or demography without knowing the other; however, none of this information is readily available, especially for non-model systems (e.g., [31,128,129,130]). Hence, Johri et al. [31] advocates treating results as viable hypotheses for future analyses, rather than proof of reality. However, a large proportion of the sweeps detected in this study overlaps with genes under selection identified by an independent cline-based approach in [16], which gives additional confidence that the putative selective sweeps detected in this study are real.

There is no perfect method and dataset that accurately detects selective sweeps. In this study, we attempted to reduce false positives by selecting software tools that are robust to demographic changes, as well as incorporating multiple supporting pieces of evidence before identifying candidates, including iHH12 and several polymorphism analyses. There may still be some false positive sweeps in our list of candidates. These form the basis for future studies, which should aim at eliminating these false positives by examining them in detail, and gathering more information about the system to construct a baseline model that is as biologically relevant as possible [31].

## 5. Conclusions

Adaptation of *Senecio* to contrasting habitats of high and low elevations on Mount Etna has been studied with many approaches (e.g., [14,16,61]), but this is the first study to carry out a genome-wide scan for selection identifying the loci and genomic regions potentially involved in adaptation, especially adaptive introgression, in *S. aethnensis* and *S. chrysanthemifolius*. Consistent with previous work on this system, we detected widespread signatures of selection in many regions across the genomes of both species. We detected over-representation of GO terms with low elevation-relevant functions in the low-altitude *S. chrysanthemifolius*. Interspecific introgression appears to play a significant role in adaptation of these species, as it is more common than expected. This may be due to their relatively small population sizes. The abundance of introgressions, associated with defence and oxidative stress, indicate the presence of common selective pressures occurring at both high and low elevations. On the other hand, a significant proportion of sweeps were species-specific and/or non-introgressive, consistent with local adaptation to the contrasting conditions at high and low elevations on Mount Etna. This work serves as a starting point for studying adaptive introgression in this recently diverged species pair. Building on similar studies of adaptive introgression in rapid adaptive radiations, conservation and crops [35,36,37,38,39,40,41,42], this study provides further insights into a previously understudied selective agent—a volcano which has tremendous influence on environmental conditions (such as the presence of xenobiotic compounds from volcanic deposits) alongside general features of an elevational gradient. Testing the ecological and adaptive values of the particular genes and mutations identified as putatively adaptive in our study will be an important future direction of work in this interesting system.

## Figures and Tables

**Table 1:** Scaffold and SNP information for each linkage group (LG). Details for polymorphism statistics can be found in Additional File: Supplementary Table S2. aeth = *S. aethnnsis*; chry = *S. chrysanthemifolius*.

LG	Scaffold No.	Length of Scaffold in Reference Genome (b)	No. of SNPs Identified	SNP Density (/b)	Mean π_syn_ (aeth)	Mean π_syn_ (chry)
1	78	68,463,240	2,398,778	0.035	0.0176	0.0154
2	612	67,696,633	2,484,653	0.037	0.0175	0.0153
3	622	52,330,192	1,757,164	0.034	0.0167	0.0141
4	619	52,882,715	1,742,806	0.033	0.0171	0.016
5	6	79,160,621	3,000,805	0.038	0.0174	0.0165
6	83	66,670,782	2,355,818	0.035	0.0179	0.0151
7	620	67,673,870	2,329,662	0.034	0.0181	0.015
8	621	57,835,927	1,910,353	0.033	0.0168	0.0143
9	538	59,368,209	2,282,327	0.038	0.0187	0.0168
10	462	59,330,977	2,199,068	0.037	0.0185	0.0169
All	All	631,413,166	22,461,434	0.036	0.0176	0.0155

## Data Availability

Raw sequence data for all *S. aethnensis* and *S. chrysanthemifolius* samples used in this study are available in GenBank under BioProject PRJNA1401787 (SRA accession no. SAMN54570847 to SAMN54570858).

## Supplementary Materials

The following supporting information can be downloaded at: https://www.mdpi.com/article/10.3390/genes17070778/s1. Figure S1: Genome-wide scans for polymorphism indices; Figure S2: Box plots comparing nucleotide diversity (π) and Tajima’s D between frequently- and rare-recombining regions in *S. aethnensis* and *S. chrysanthemifolius* respectively; Figure S3: Correlation between gene count per 10Mb and number of different types of sweeps (non-introgressive and introgressive) in each species; Figure S4: Genome-wide plots of various polymorphism indices and scans of introgressive and positive sweeps and for each linkage group (LG); Figure S5: Recombination landscape in LG 1–10; Figure S6: Over-represented GO terms related to biological processes in introgressive sweeps in *S. chrysanthemifolius*; Figure S7: Over-represented GO terms related to molecular function in introgressive sweeps in *S. chrysanthemifolius*; Figure S8: Over-represented GO terms related to cellular components in introgressive sweeps in *S. chrysanthemifolius*. Table S1: Information of the samples used in this study. All sequences are deposited under the GenBank BioProject PRJNA1401787; Table S2: Mean polymorphism statistics in each LG. Abbreviations: syn = synonymous sites, nsyn = non-synonymous sites, ncd = non-coding sites, all = all sites, aeth = *S. aethnensis*, chry = *S. chrysanthemifolius*; Table S3: Results of Kruskal-Wallis test comparing various polymorphism statistics between *S. aethnensis* and *S. chrysanthemifolius*. df = 1 for all comparisons; Table S4: Positive selective sweep regions detected by RAiSD supported by iHH12 analysis; Table S5: Introgression sweep regions detected by VolcanoFinder supported by iHH12 analysis; Table S6: Number and length of selective sweeps detected by VolcanoFinder and RAiSD in *S. aethnensis* and *S. chrysanthemifolius*, and details about shared sweep regions between the two softwares in each species (>10 kb overlap in sweep regions); Table S7: Number of genes queried with The Gene Ontology Resource (http://geneontology.org/) and the number of unmapped gene IDs, using *Arabidopsis thaliana* as reference; Table S8: List of over-represented GO terms in local introgressive sweeps in *S. chrysnathemifolius*; Table S9: Selective coefficients of previousy-identified high-differentiated markers, re-estimated in this study using output from VolcanoFinder and from reference [16].

## Abbreviations

The following abbreviations are used in this manuscript:

SNP	Single-nucleotide polymorphism
LG	Linkage groups
CLR	Composite likelihood ratio
GAM	Generalised additive model
UV	Ultraviolet light
GO	Gene Ontology
