# Supplementary material for: Adaptative Introgression and Local Adaptation Sweep Through High- and Low-Elevation Senecio Species on Mount Etna, Sicily
Source: Genes (Basel). 2026 Jul 1;17(7):778. doi: 10.3390/genes17070778 (PMC13410233; doi:10.3390/genes17070778)
Supplement: Supplementary file 1 [file genes-17-00778-s001.zip › genes-4377644-supplementary.pdf]

Supplementary Figures

Adaptative introgression and local adaptation sweep through high- and low-elevation Senecio on Mount Etna, Sicily

Edgar L.Y. Wong[1,2,*], Simon J. Hiscock[1,3] and Dmitry A. Filatov[1,*]

[1] Department of Biology, University of Oxford, Oxford, UK
[2] Senckenberg – Leibniz Institution for Biodiversity and Earth System Research, Senckenberg Biodiversity and Climate Research Centre, Frankfurt am Main, Germany
[3] Oxford Botanic Garden and Arboretum, Oxford, UK
* Corresponding authors: Edgar L.Y. Wong: edgar.wong@senckenberg.de; Dmitry A. Filatov: dmitry.filatov@biology.ox.ac.uk

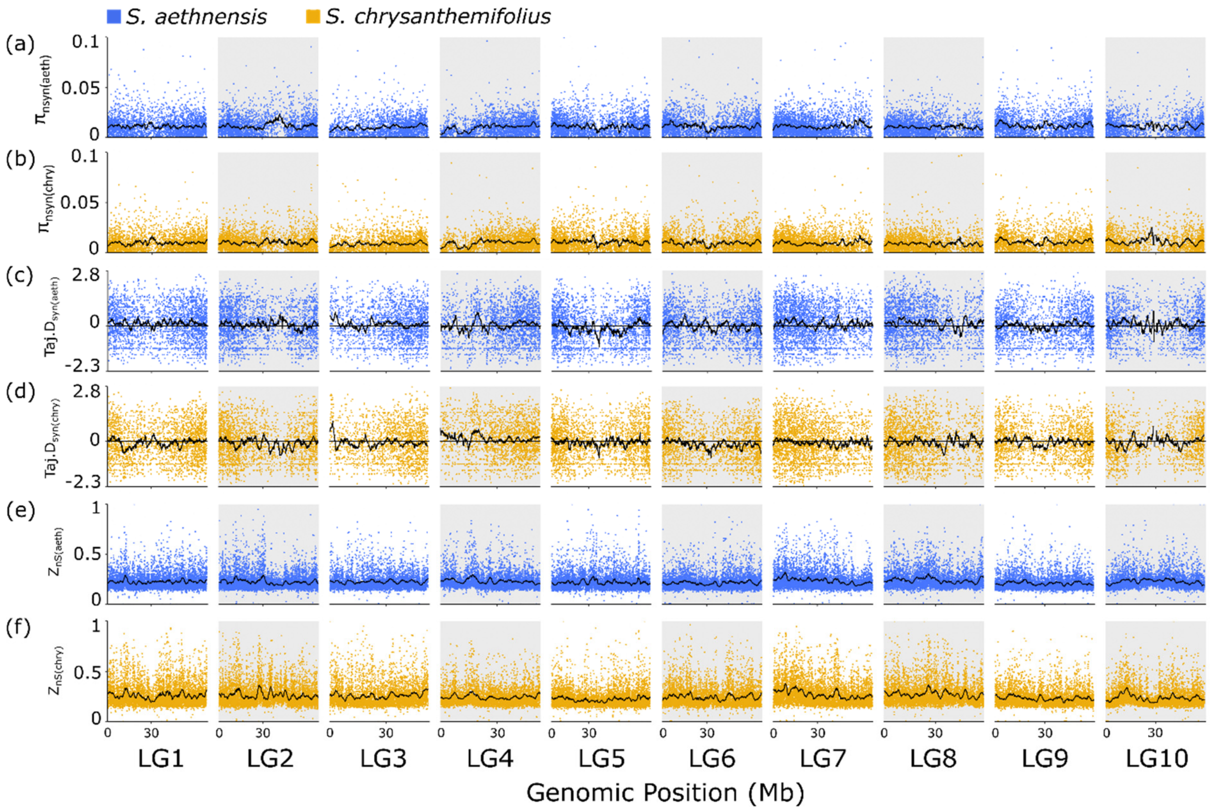

Supp. Figure S1. Genome-wide scans for polymorphism indices. Plots from top to bottom are (**a-b**) nucleotide diversity (π) (non-synonymous coding sites) for S. aethnensis and S. chrysanthemifolius; (**c-d**) Tajima's D (synonymous coding sites) for each species; (**e-f**) ZnS (all sites) for each species. All windows are non-overlapping 10kb. Black lines in (**a**) to (**f**) denote moving average of every 200 consecutive windows.

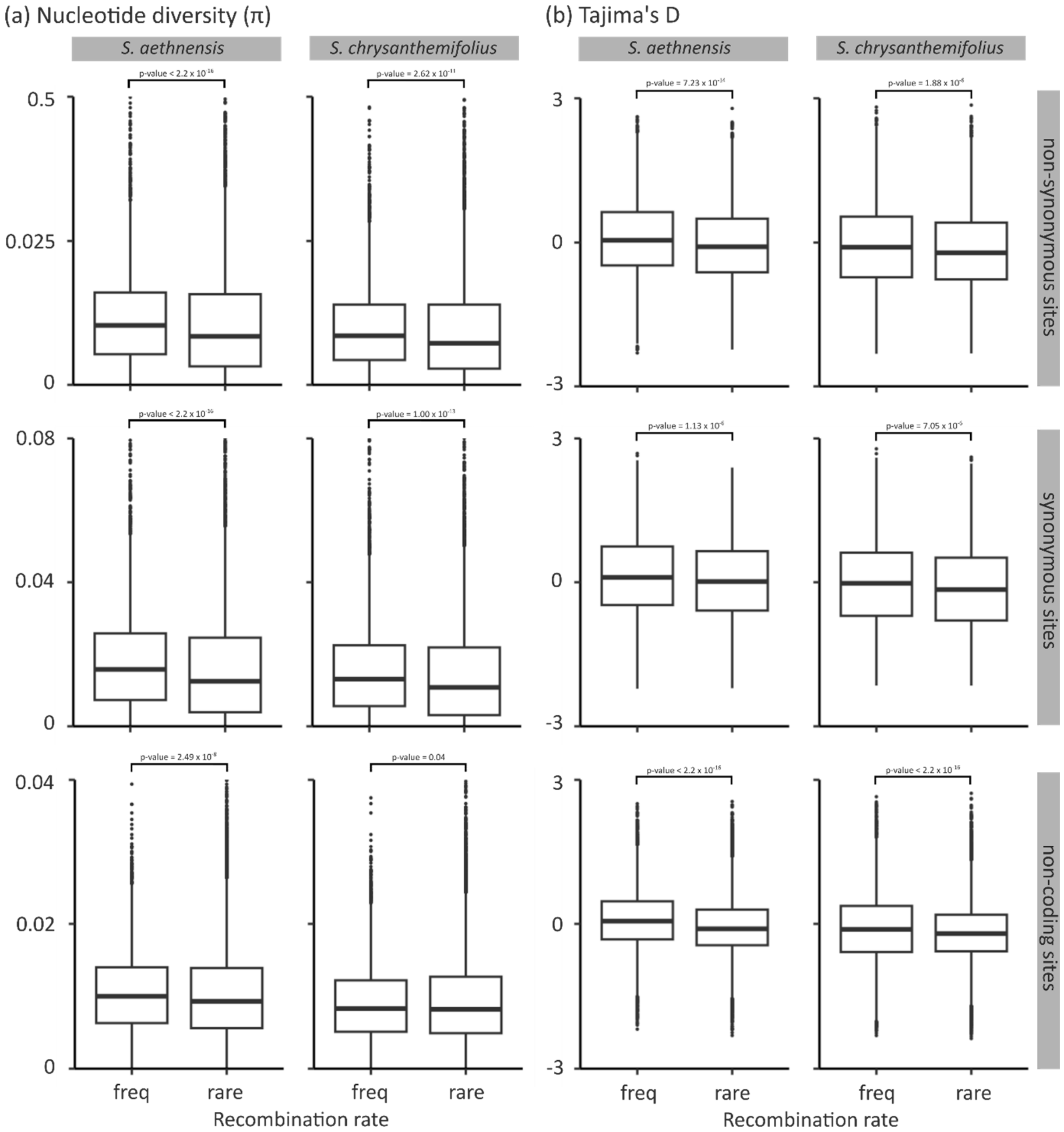

Supp. Figure S2. Box plots comparing nucleotide diversity (π) and Tajima's D between frequently- and rare-recombining regions in *S. aethnensis* and *S. chrysanthemifolius* respectively. For each index, non-synonymous sites, synonymous sites and non-coding sites were compared. Outliers exceeding π = 0.5, 0.08 and 0.04 for synonymous, non-synonymous and non-coding sites respectively were not shown to increase resolution in figure.

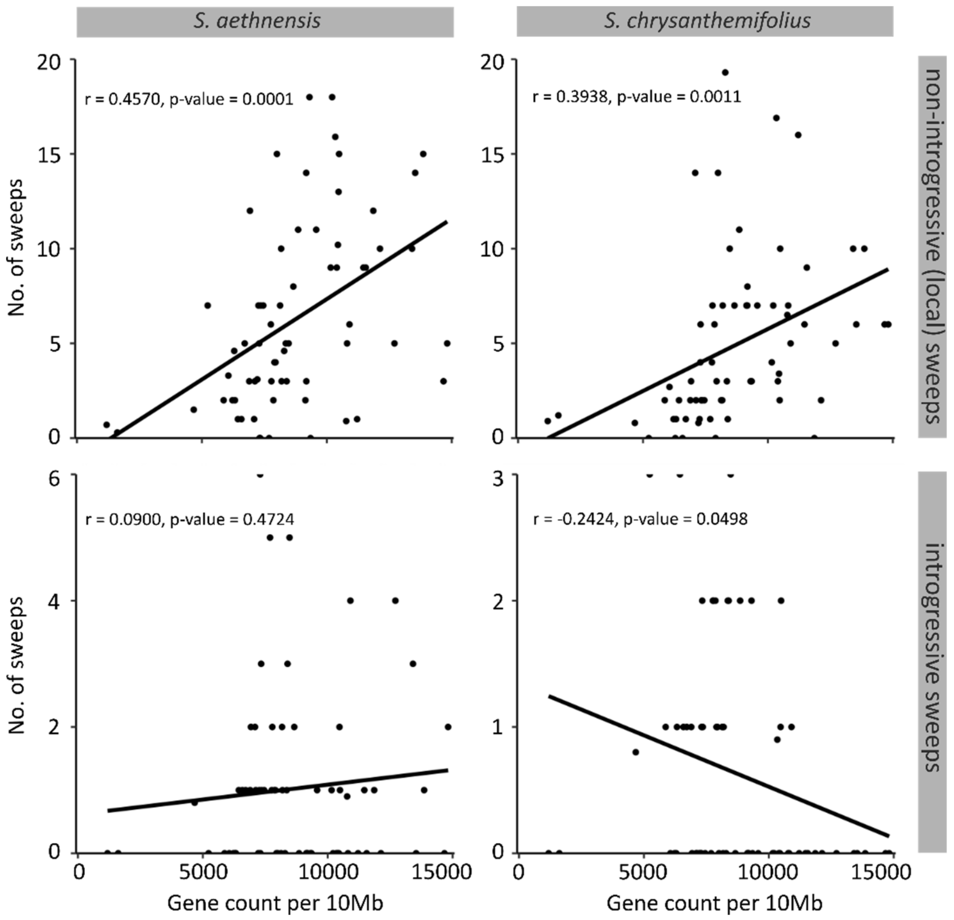

Supp. Figure S3. Correlation between gene count per 10Mb and number of different types of sweeps (non-introgressive and introgressive) in each species.

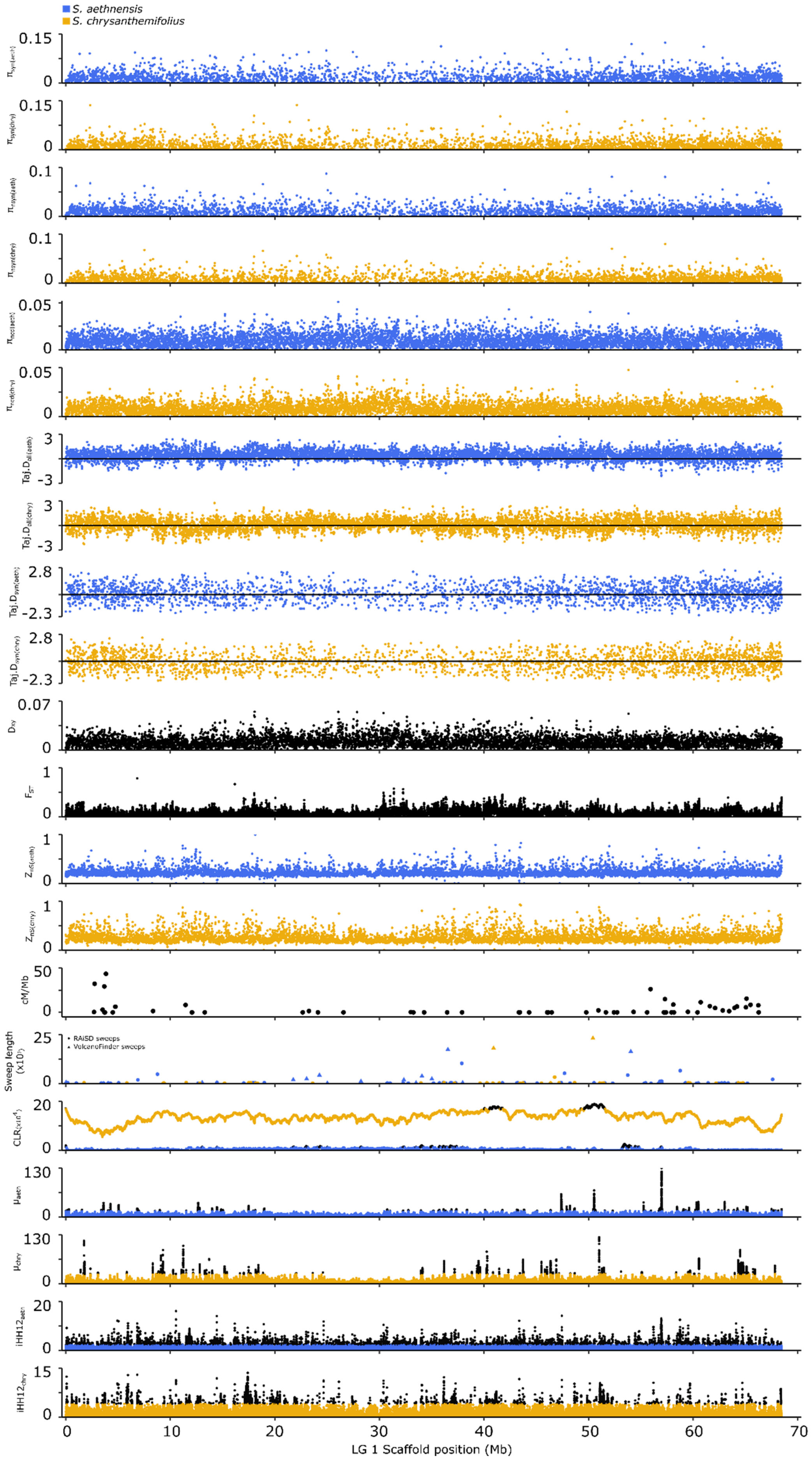
S. aethnensis
S. chrysanthemifolius
Taj.D
Dxy
FST
cM/Mb
Sweep length
RAiSD sweeps
VolcanoFinder sweeps
CLR
iHH12
LG 1 Scaffold position (Mb)

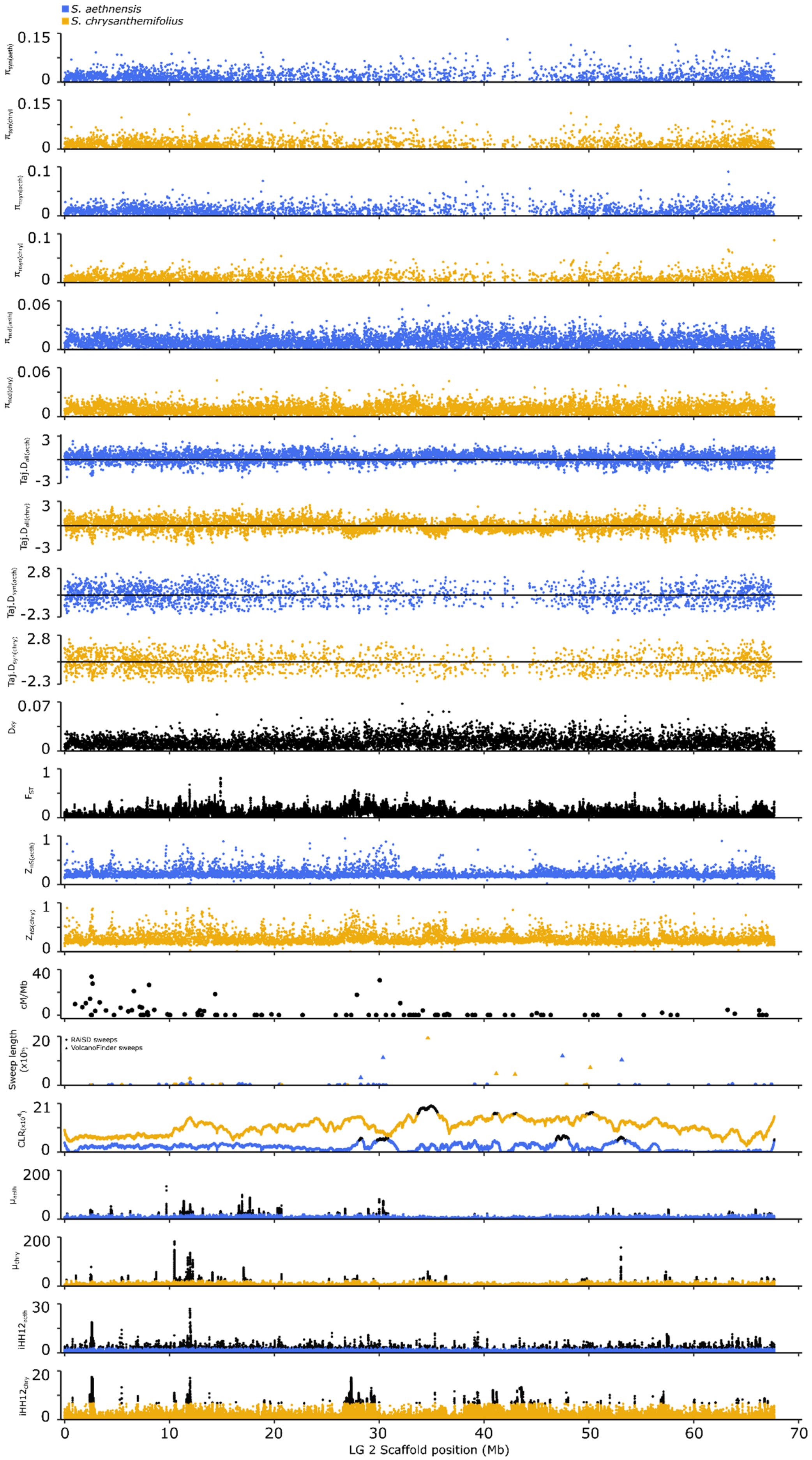

S. aethnensis
S. chrysanthemifolius
RAiSD sweeps
VolcanoFinder sweeps
Sweep length
cM/Mb
CLR
Dxy
FST
iHH12
0
10
20
30
40
50
60
70
LG 2 Scaffold position (Mb)

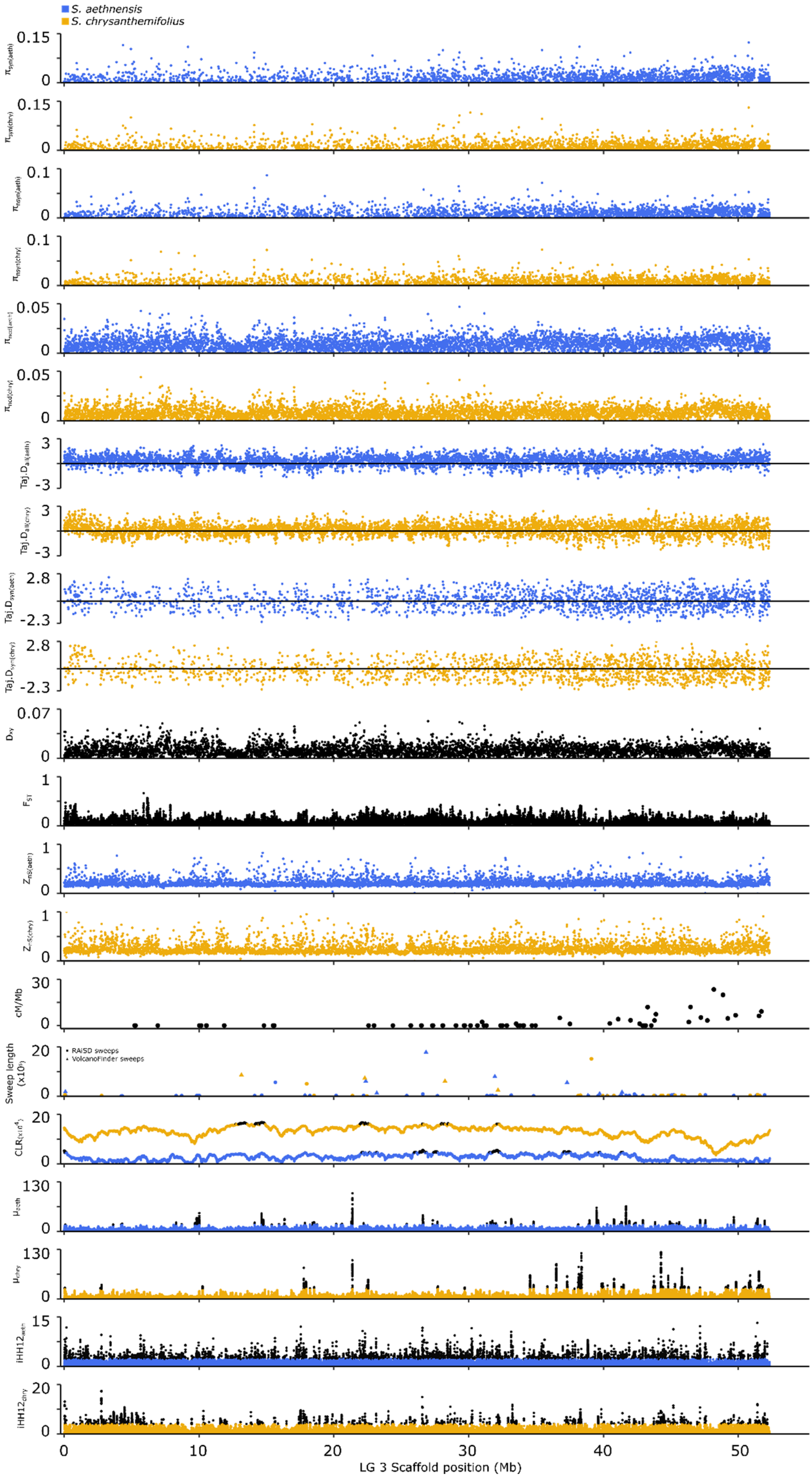

S. aethnensis
S. chrysanthemifolius
Sweep length (x10⁵)
RAiSD sweeps
VolcanoFinder sweeps
CLR (x10⁴)
LG 3 Scaffold position (Mb)

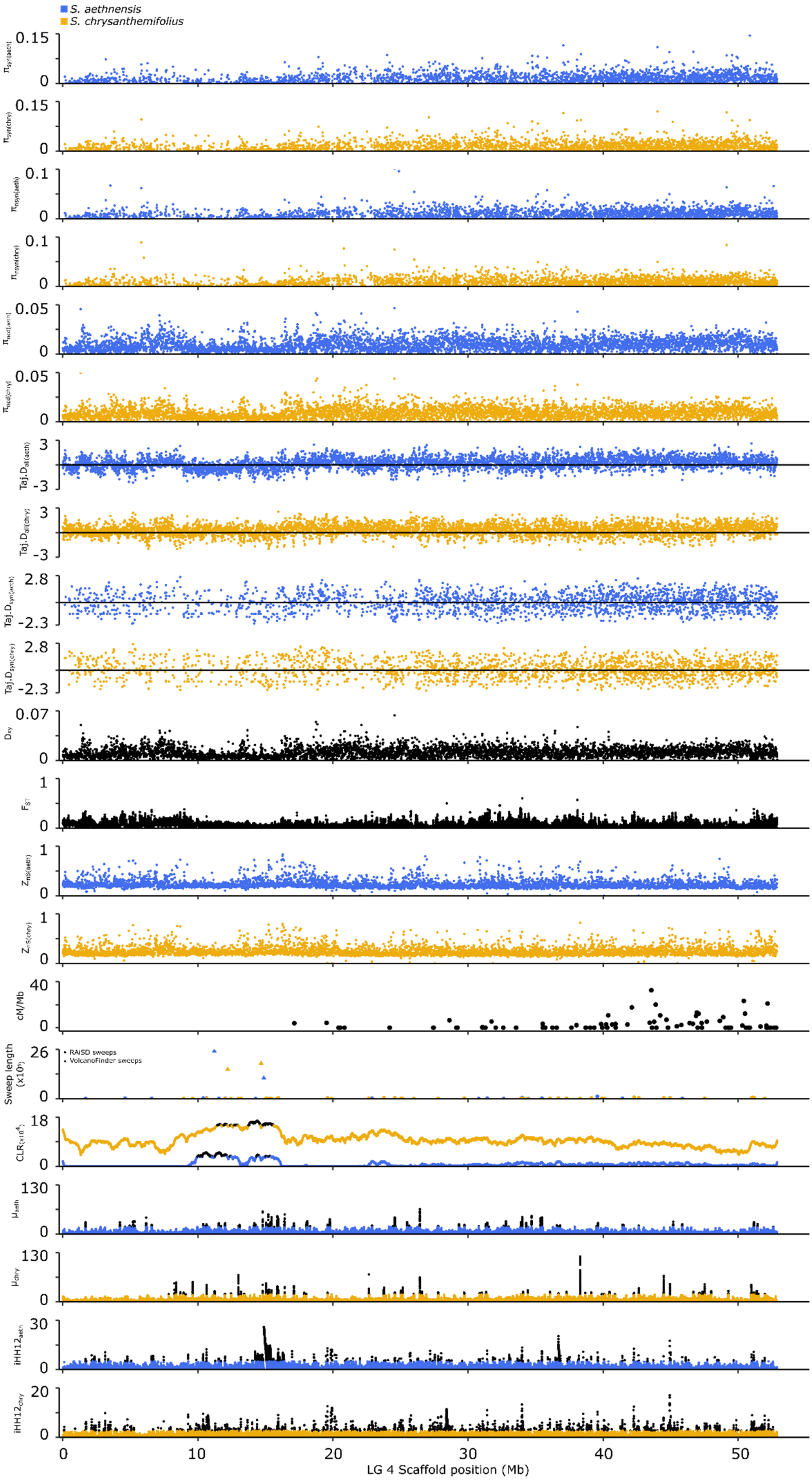

S. aethnensis
S. chrysanthemifolius
cM/Mb
Sweep length (x10⁵)
RAiSD sweeps
VolcanoFinder sweeps
0
10
20
30
40
50
LG 4 Scaffold position (Mb)

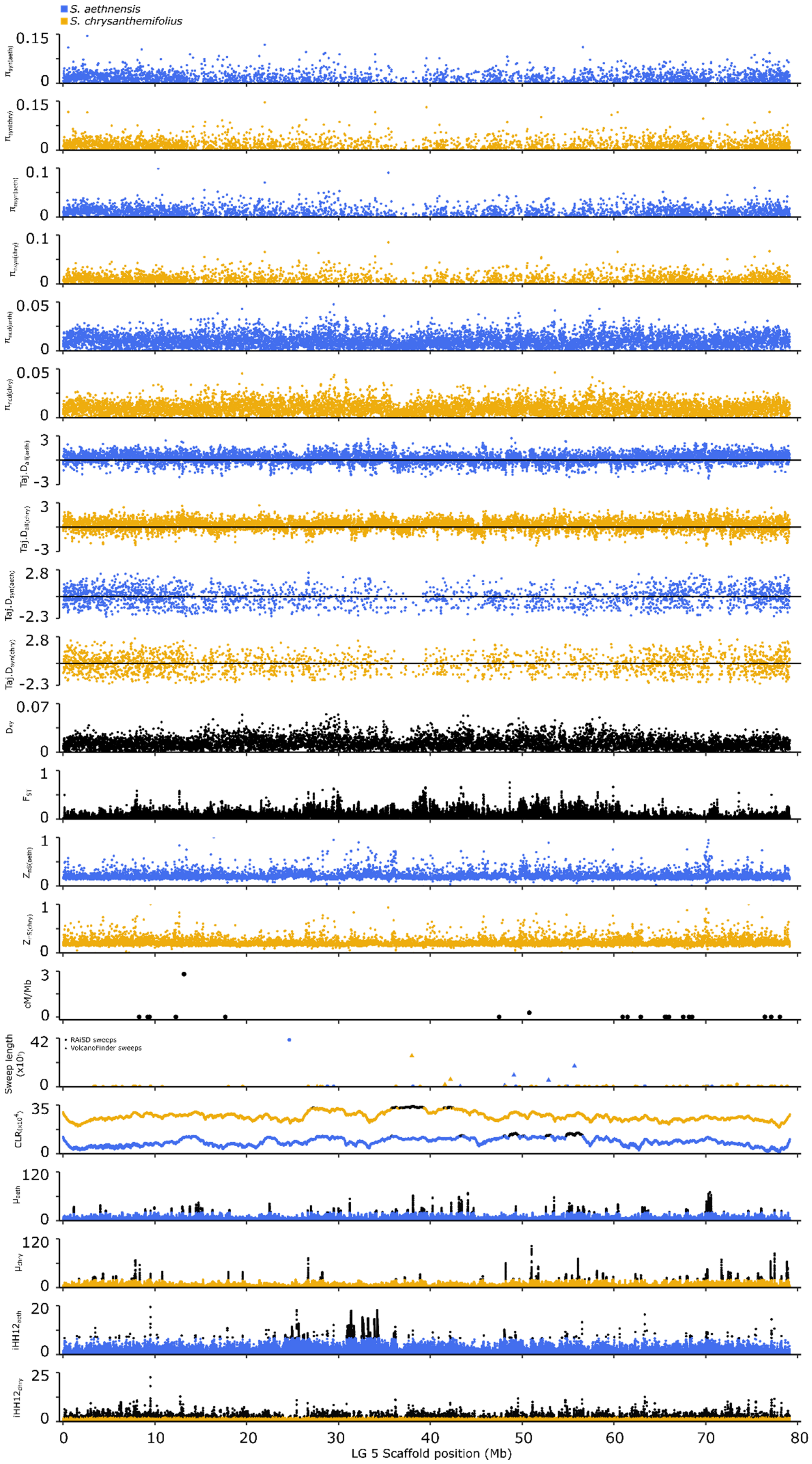

S. aethnensis
S. chrysanthemifolius
0.15
0.1
0.05
3
-3
2.8
-2.3
0.07
1
0
42
35
120
20
25
RAiSD sweeps
VolcanoFinder sweeps
Sweep length
CLR
cM/Mb
Dxy
FST
0
10
20
30
40
50
60
70
80
LG 5 Scaffold position (Mb)

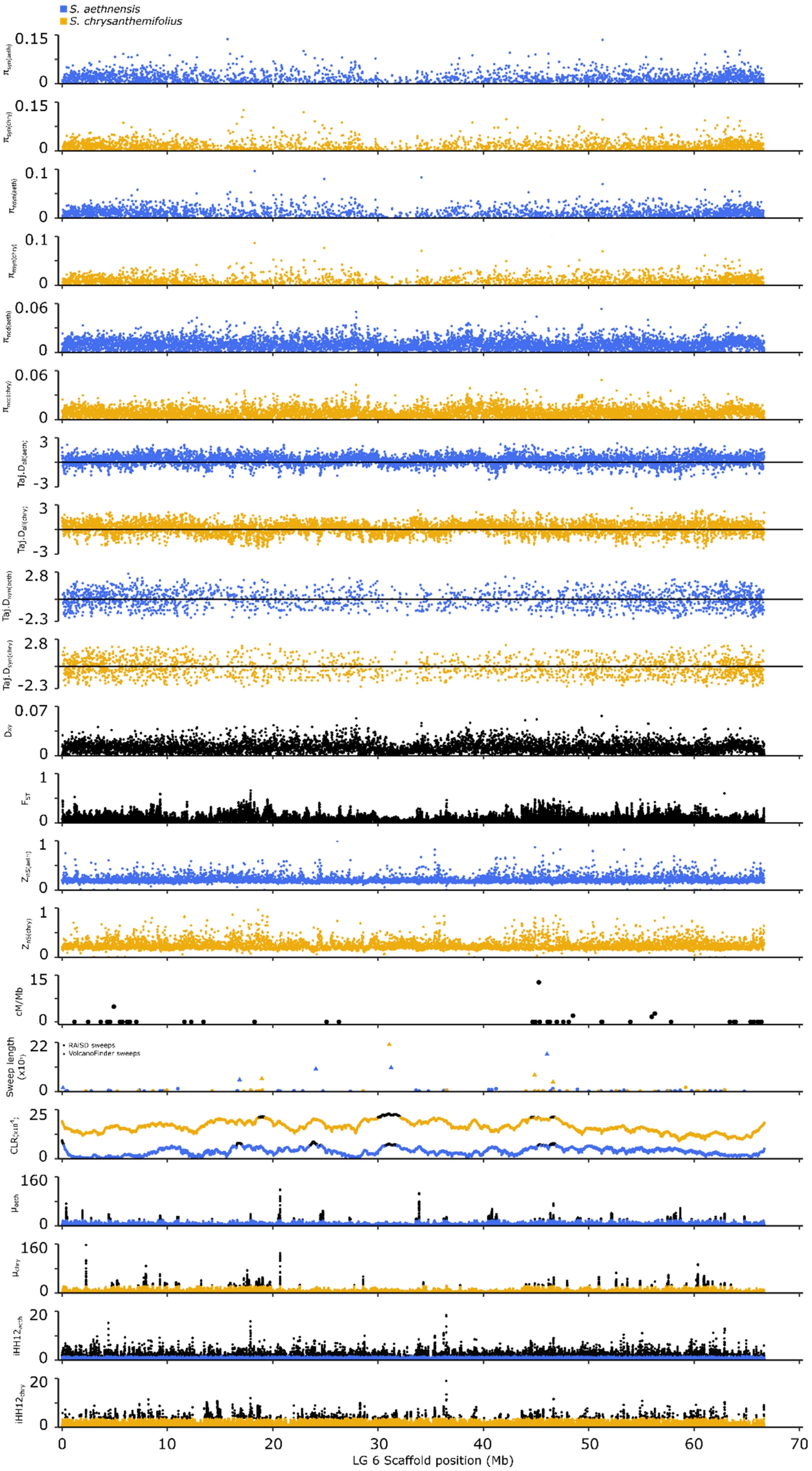

S. aethnensis
S. chrysanthemifolius
0.15
0
0.1
0.06
3
-3
2.8
-2.3
0.07
D_{xy}
1
F_{ST}
15
cM/Mb
22
Sweep length
RAISD sweeps
VolcanoFinder sweeps
25
160
20
0
10
20
30
40
50
60
70
LG 6 Scaffold position (Mb)

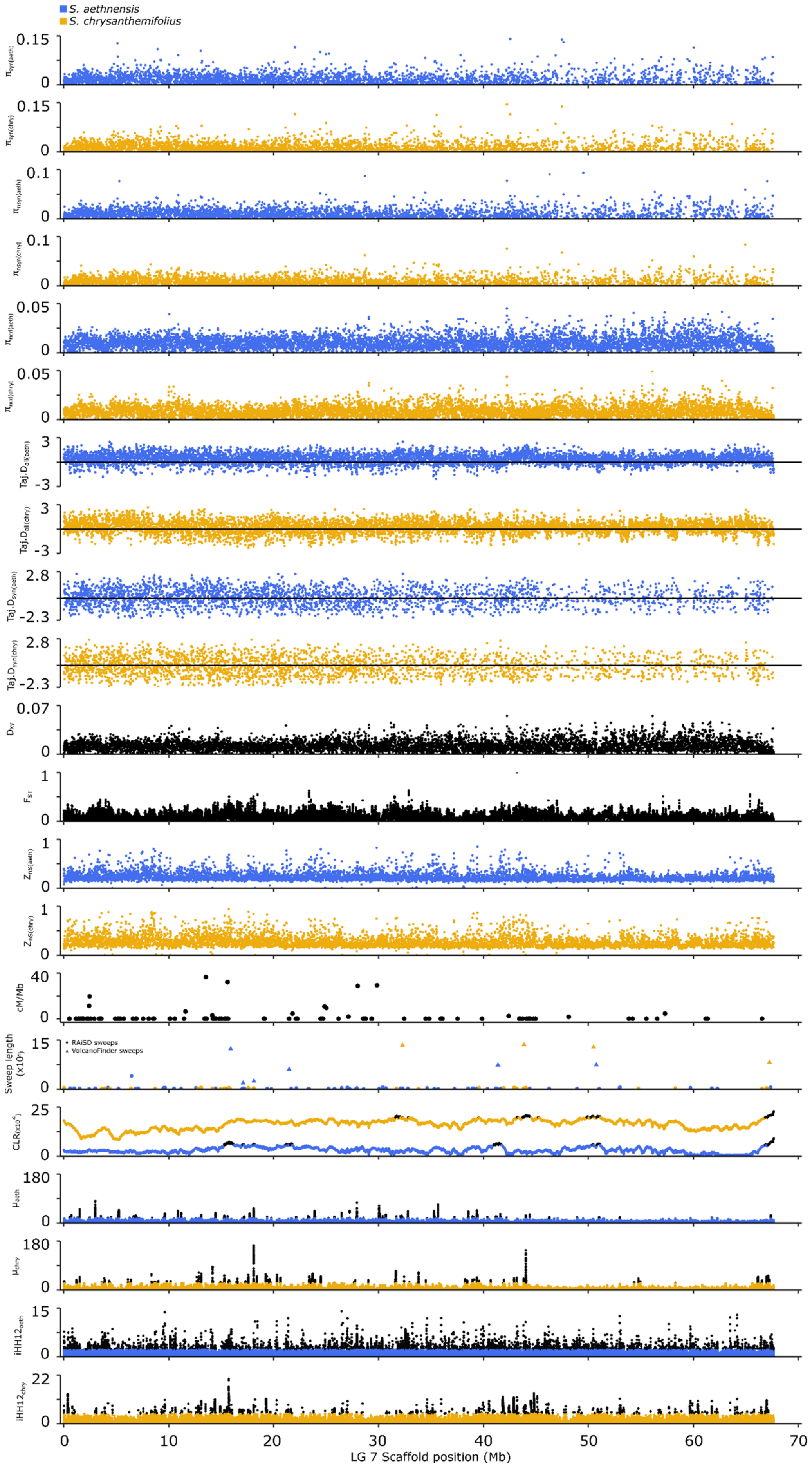

S. aethnensis
S. chrysanthemifolius
RAiSD sweeps
VolcanoFinder sweeps
LG 7 Scaffold position (Mb)

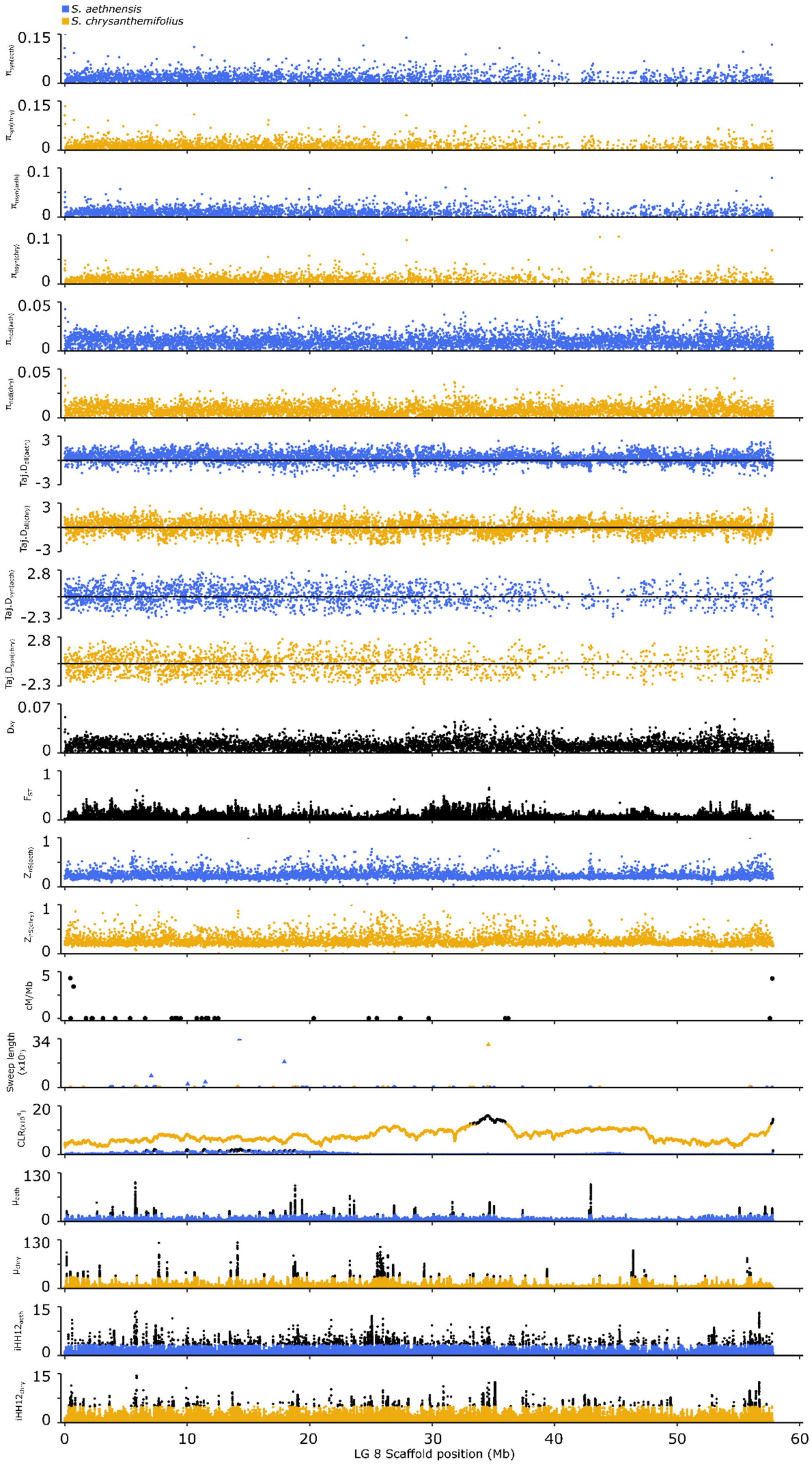
S. aethnensis
S. chrysanthemifolius
Taj.D
D_{xy}
F_{ST}
cM/Mb
Sweep length
CLR
iHH12
0
10
20
30
40
50
60
LG 8 Scaffold position (Mb)

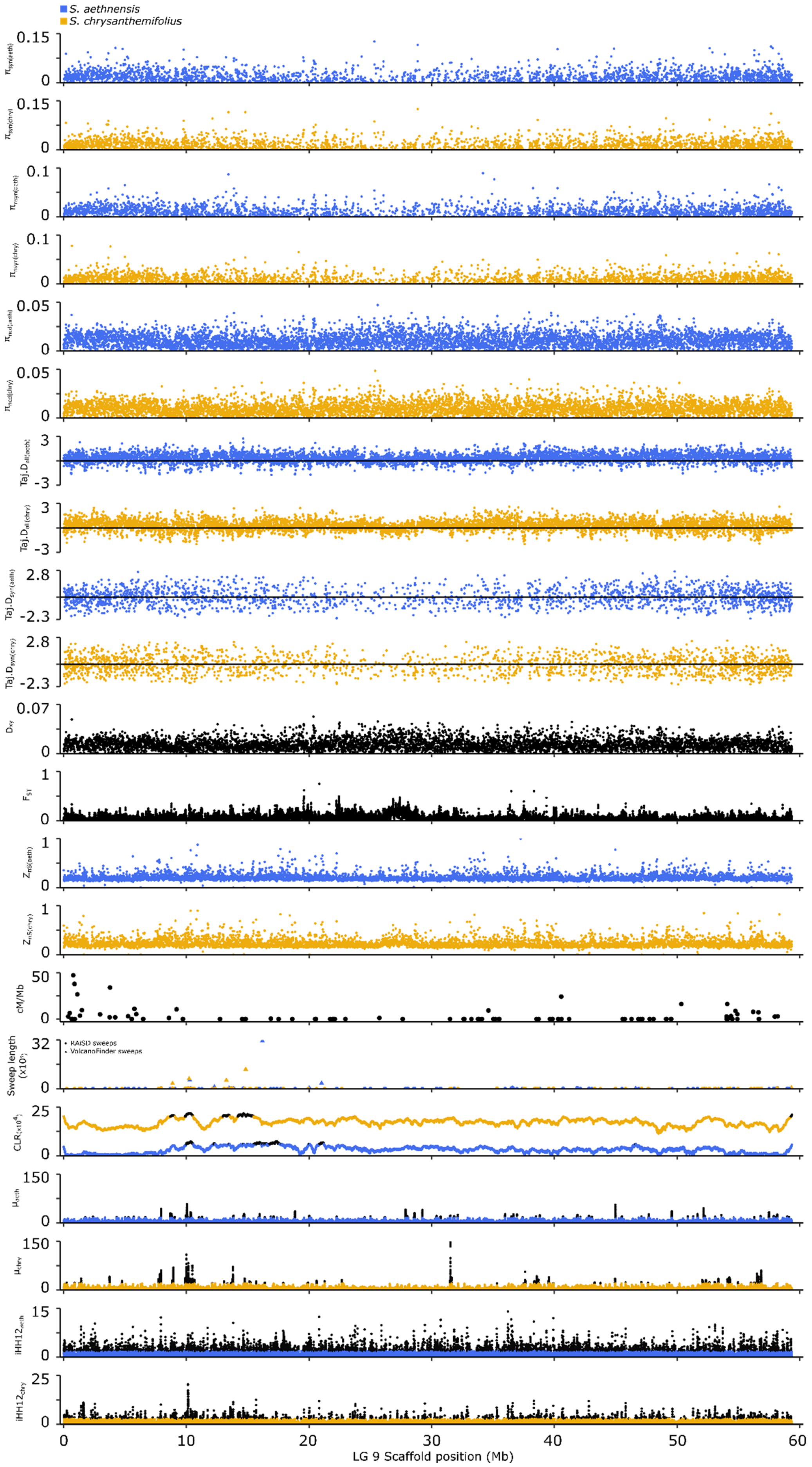

S. aethnensis
S. chrysanthemifolius
cM/Mb
Sweep length
RAiSD sweeps
VolcanoFinder sweeps
0
10
20
30
40
50
60
LG 9 Scaffold position (Mb)

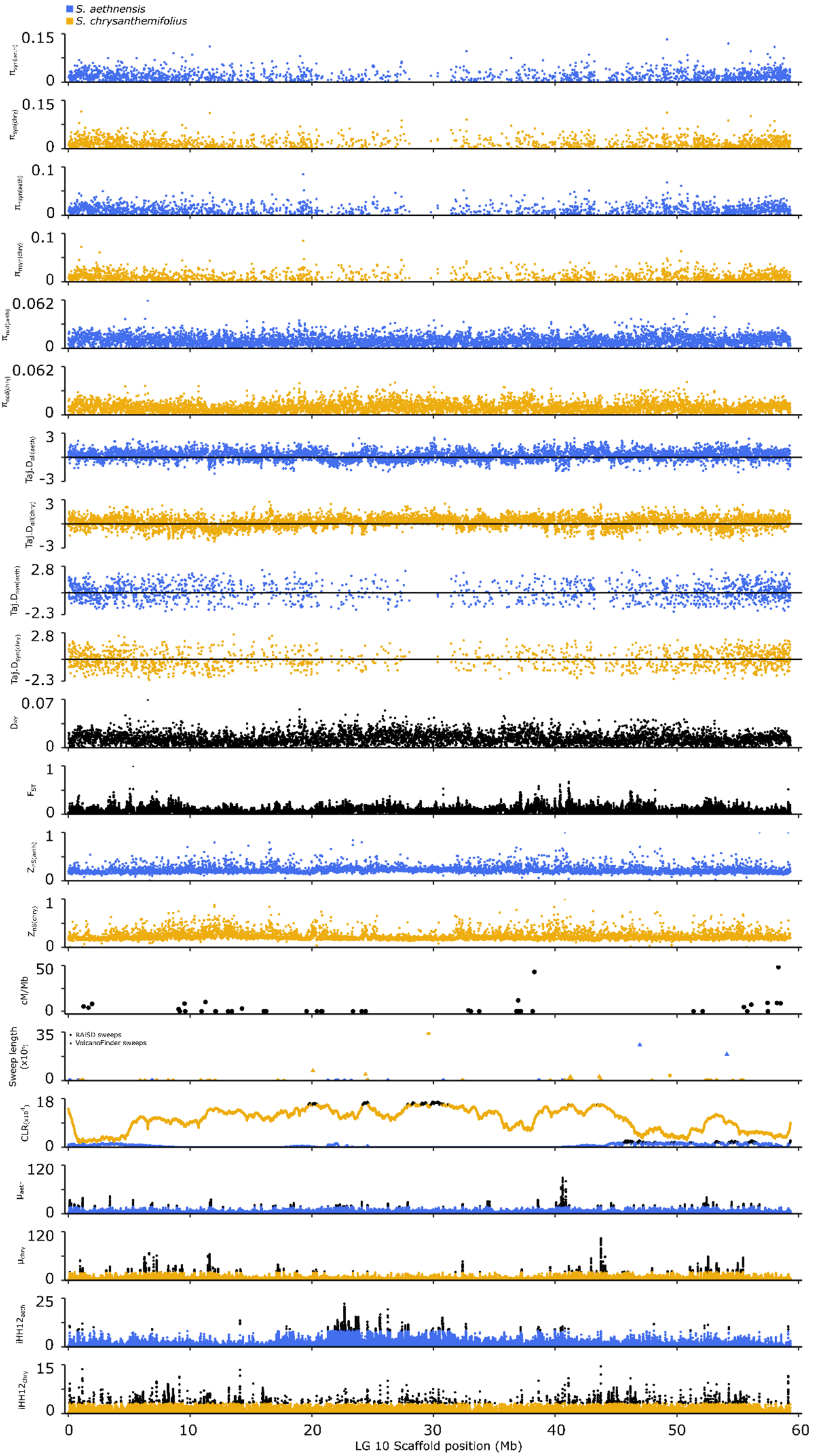

Supp. Figure S4. Genome-wide plots of various polymorphism indices and scans of introgressive and positive

sweeps and for each linkage group (LG). Plots from top to bottom for each LG (one page each) are: genome scan for nucleotide diversity (π) for *S. aethnensis* and *S. chrysanthemifolius* using synonymous (π_{syn}), non-synonymous (π_{nsyn}) and non-coding (π_{ncd}) sites respectively, Tajima's D for each species using all (Taj.D_{all}) and synonymous (Taj.D_{syn}) sites respectively, D_{xy} between both species, F_{ST} between both species, Z_{nS} for each species, recombination rate, distribution of length of sweeps, composite likelihood ratio (CLR0 from scans of introgressive sweeps using VolcanoFinder for each species, μ statistic from scans of positive sweeps using RAiSD for each species, and normalised iHH12 values for each species. F_{ST} plots consist of overlapping windows of 10kb (2kb step). π, Tajima's D, D_{xy} and Z_{nS} plots consist of non-overlapping windows of 10 kb. π_{syn}>0.15 and π_{nsyn}>0.1 are excluded: for π_{syn}, a total of 33 and 29 windows were excluded for *S. aethnensis* and *S. chrysanthemifolius* respectively, whereas for $\pi_{nsy,}$ 25 windows were excluded for each species. In VolcanoFinder, test sites were present every 1 kb, with top 5% of data points in each LG treated as outliers (black). Sliding window size (in terms of number of SNPs) in RAiSD plots is 100, with top 1% of data points in each LG treated as outliers (black). iHH12 windows size is determined by default in *selscan*.

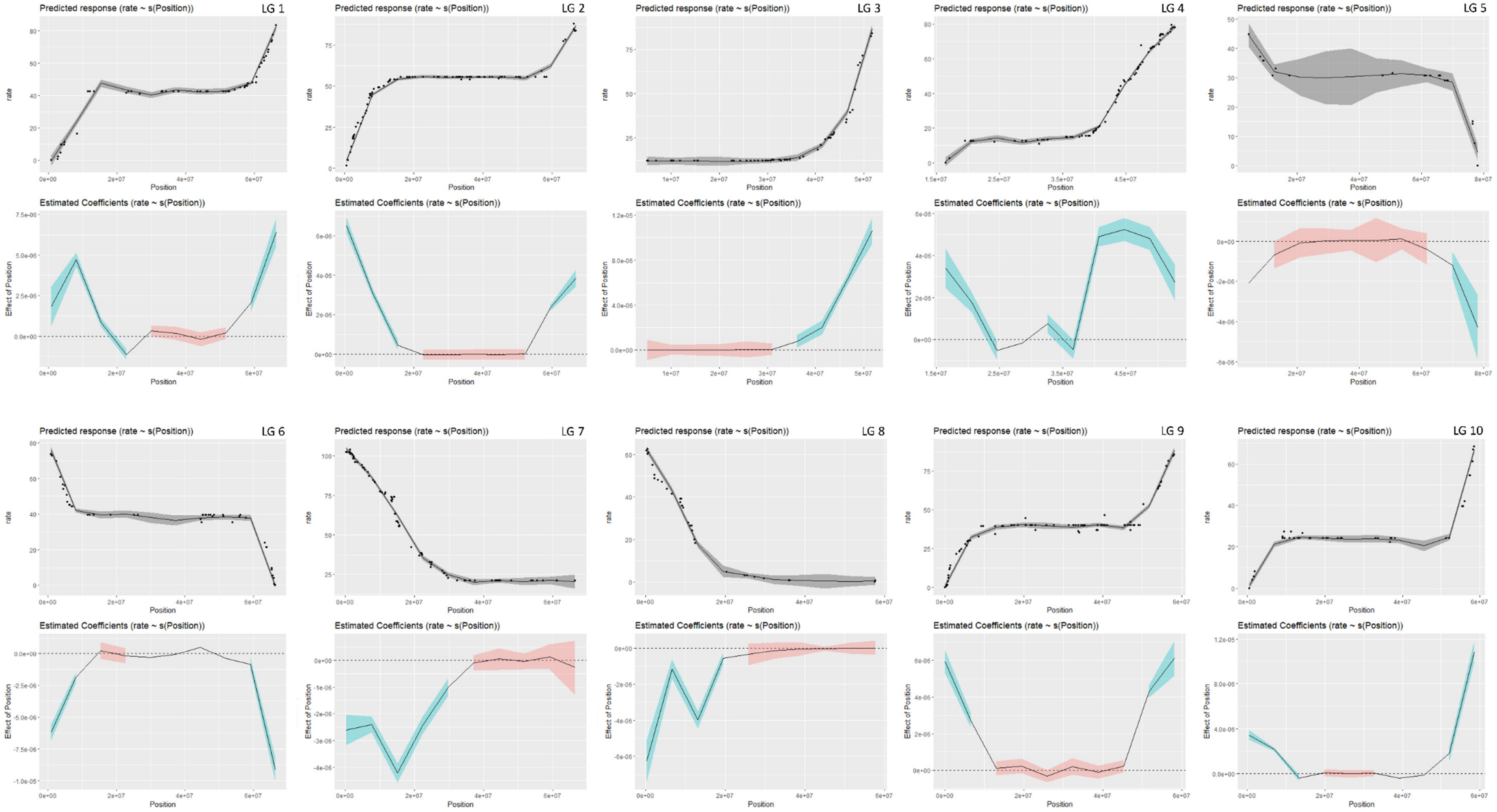

Supp. Figure S5. Recombination landscape in LG 1 -10. For each LG, the top figure is the fitted curve of recombination rate using the generalized additive model; the bottom figure is the derivative of the curve. rate = estimated recombination rate using markers from reference [61]; Position = genomic position on each LG.

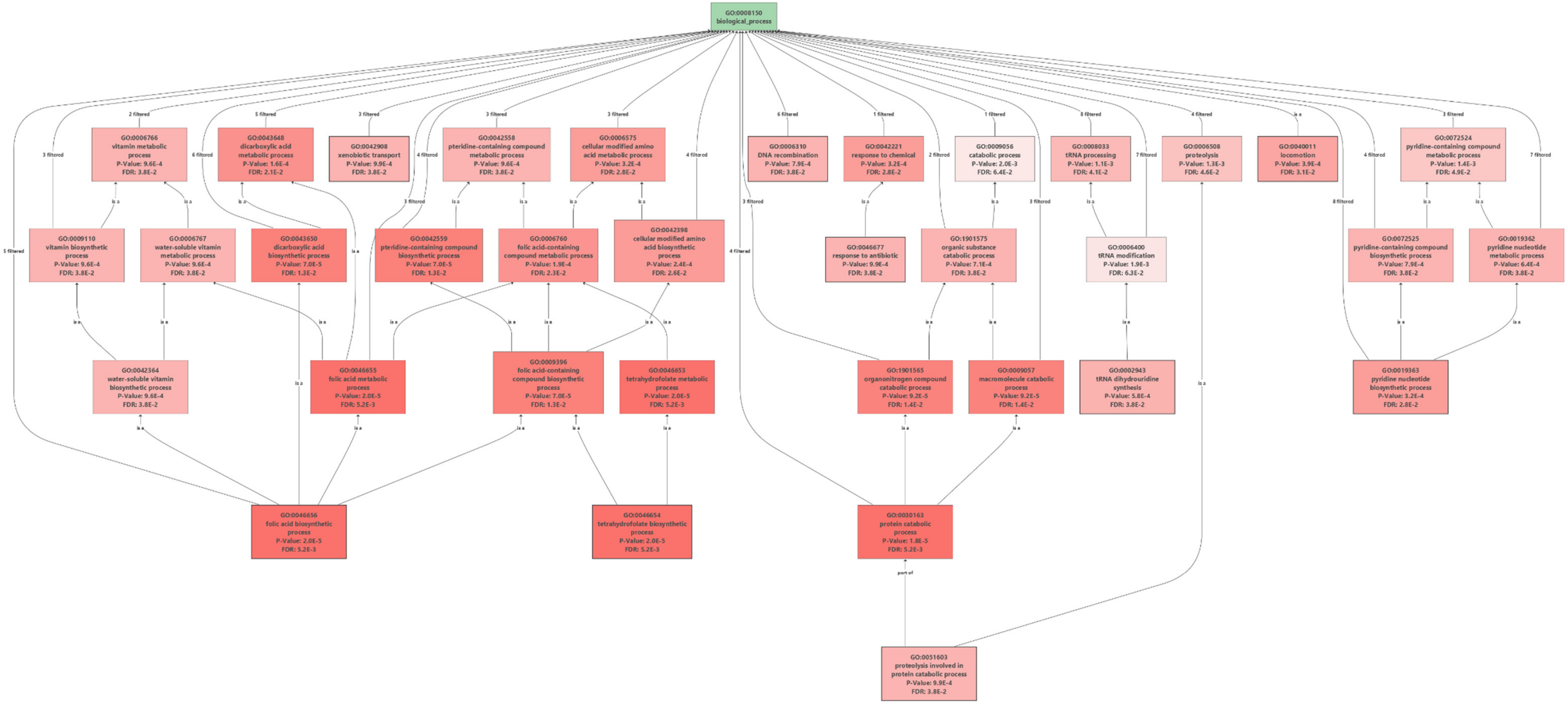

Supp. Figure S6. Over-represented GO terms related to biological processes in introgressive sweeps in *S. chrysanthemifolius*.

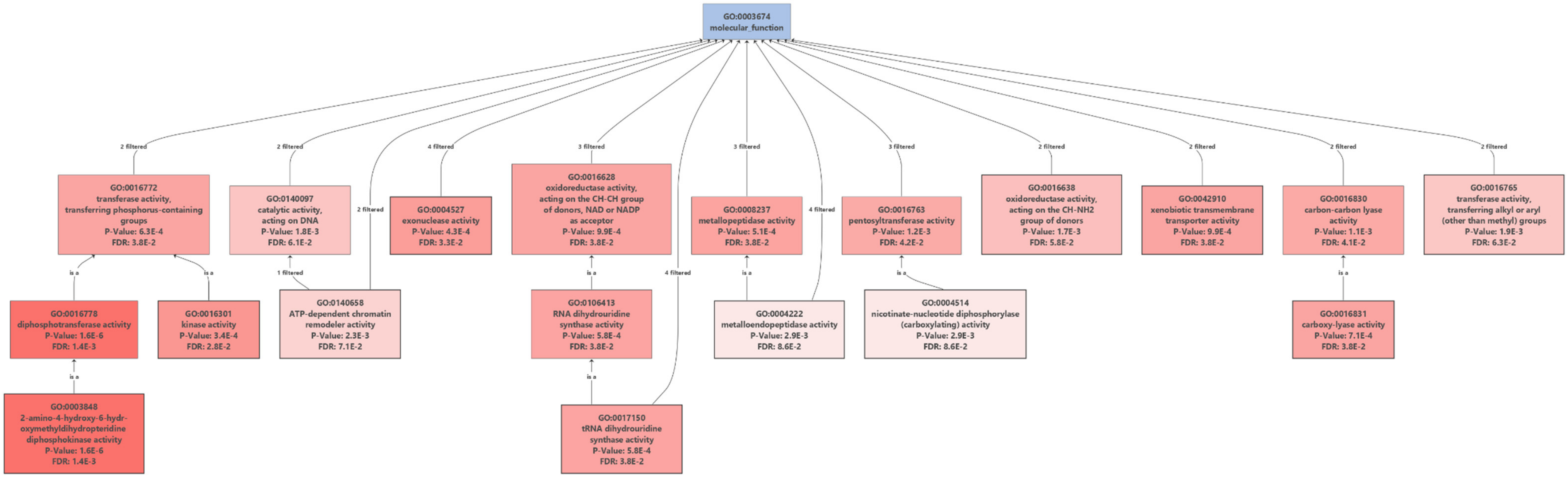

Supp. Figure S7. Over-represented GO terms related to molecular function in introgressive sweeps in *S. chrysanthemifolius*.

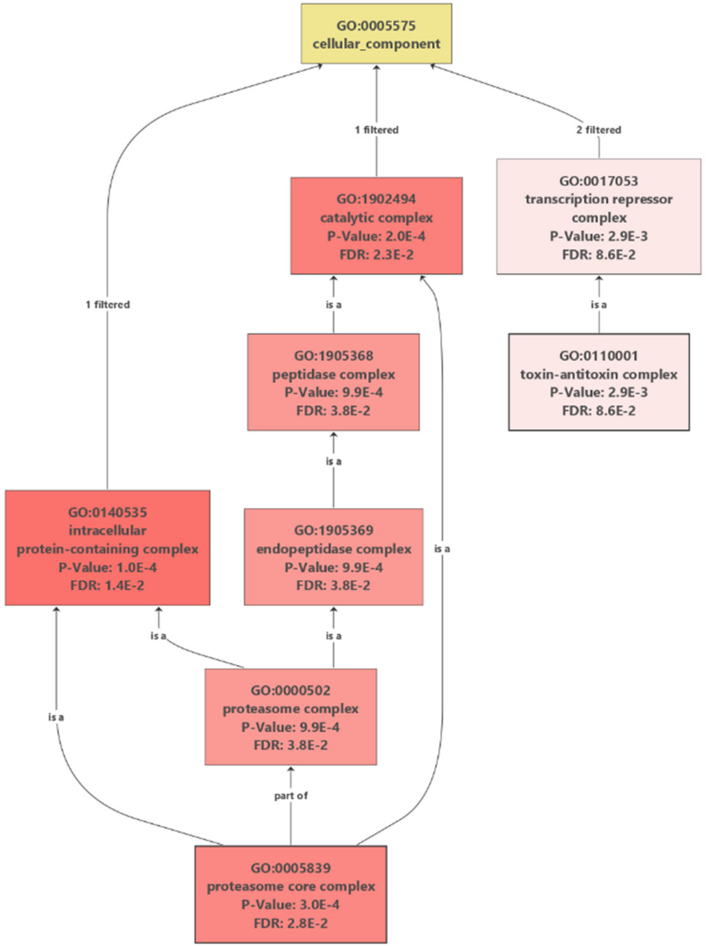

Supp. Figure S8. Over-represented GO terms related to cellular components in introgressive sweeps in *S. chrysanthemifolius*.
